# Delivering Mindfulness-Based Interventions for Insomnia, Pain, and Dysfunctional Eating Through a Text Messaging App: Three Randomized Controlled Trials Investigating the Effectiveness and Mediating Mechanisms

**DOI:** 10.2196/30073

**Published:** 2022-05-03

**Authors:** Amanda CM Li, Keith KL Wong, Floria HN Chio, Winnie WS Mak, Loretta WH Poon

**Affiliations:** 1 Department of Psychology The Chinese University of Hong Kong Hong Kong Hong Kong; 2 Clinical Psychological Services New Life Psychiatric Rehabilitation Association Hong Kong Hong Kong; 3 Department of Counselling and Psychology Hong Kong Shue Yan University Hong Kong Hong Kong; 4 newlife.330 New Life Psychiatric Rehabilitation Association Hong Kong Hong Kong

**Keywords:** text messaging, mindfulness, insomnia, pain, dysregulated eating, mHealth, mental health, SMS, distress, intervention, outcome, mobile interventions

## Abstract

**Background:**

Although text messaging has the potential to be the core intervention modality, it is often used as an adjunct only. To improve health and alleviate the distress related to insomnia, pain, and dysregulated eating of people living in urban areas, text messaging–based mindfulness-based interventions were designed and evaluated in 3 randomized controlled trials.

**Objective:**

This study investigated the effectiveness and mediating mechanisms of text messaging–based mindfulness-based interventions for people with distress related to insomnia, pain, or dysregulated eating.

**Methods:**

In these trials, 333, 235, and 351 participants were recruited online and randomized to intervention and wait-list control conditions for insomnia, pain, and dysregulated eating, respectively. Participants experienced 21 days of intervention through WhatsApp Messenger. Participants completed pre-, post-, 1-month follow-up, and 3-month follow-up self-report questionnaires online. The retention rates at postmeasurements were 83.2% (139/167), 77.1% (91/118), and 72.9% (129/177) for intervention groups of insomnia, pain, and dysregulated eating, respectively. Participants’ queries were answered by a study technician. Primary outcomes included insomnia severity, presleep arousal, pain intensity, pain acceptance, and eating behaviors. Secondary outcomes included mindfulness, depression, anxiety, mental well-being, and functional impairments. Mindfulness, dysfunctional beliefs and attitudes about sleep, pain catastrophizing, and reactivity to food cues were hypothesized to mediate the relationship between the intervention and outcomes.

**Results:**

For all 3 studies, the intervention groups showed significant improvement on most outcomes at 1-month follow-up compared to their respective wait-list control groups; some primary outcomes (eg, insomnia, pain, dysregulated eating indicators) and secondary outcomes (eg, depression, anxiety symptoms) were sustained at 3-month follow-up. Medium-to-large effect sizes were found at postassessments in most outcomes in all studies. In the intervention for insomnia, mediation analyses showed that dysfunctional beliefs and attitudes about sleep mediated the effect of the intervention on all primary outcomes and most secondary outcomes at both 1-month and 3-month follow-ups, whereas mindfulness mediated the intervention effect on presleep arousal at 1-month and 3-month follow-ups. In the intervention for pain, pain catastrophizing mediated the effect of intervention on pain intensity and functioning at both 1-month and 3-month follow-ups, whereas mindfulness only mediated the effect of intervention on anxiety and depressive symptoms. In the intervention for dysregulated eating, power of food mediated the effect of intervention on both uncontrolled and emotional eating at both 1-month and 3-month follow-ups and mindfulness was found to mediate the effect on depressive symptoms at both 1-month and 3-month follow-ups.

**Conclusions:**

These 3 studies converged and provided empirical evidence that mindfulness-based interventions delivered through text messaging are effective in improving distress related to sleep, pain, and dysregulated eating. Text messaging has the potential to be a core intervention modality to improve various common health outcomes for people living a fast-paced lifestyle.

**Trial Registration:**

Clinical Research and Biostatistics Clinical Trials Registry CUHK_CCRB00559; https://tinyurl.com/24rkwarz

## Introduction

### Prevalence of Insomnia, Pain, and Dysregulated Eating

People living in urban areas have prevailing complaints of stress and related health concerns. For instance, the general population of Hong Kong has a stressful life, with an estimation of 13.3% having common mental disorders [1]. Stress was found to be correlated with multiple health issues, including insomnia, chronic pain, and unhealthy eating behaviors [2-4]. In the United Kingdom, the economic burden of low back pain, insomnia, and eating-related conditions was estimated to be £2.79 billion, £46.3 billion, and £8.5 billion, respectively (US $1=£0.77), projected into 2018 costs [5-7]. In Hong Kong, insomnia and chronic pain affect 35.2% and 39.4% of the general population, respectively, and close to half (46.9%) of the general population was found to have unhealthy eating habits [8-10].

### Mindfulness-Based Interventions for Insomnia, Pain, and Dysregulated Eating

To tackle these health concerns, mindfulness-based interventions (MBIs) have been applied to alleviate stress and enhance well-being. Mindfulness is defined as “paying attention in a particular way on purpose in the present moment and nonjudgmentally” [11]. In particular, MBIs were found to be effective in improving insomnia [12,13], pain [14-16], binge eating, and emotional eating [17,18]. MBIs have not only been applied to clinical populations, but they have also been demonstrated to be beneficial to the well-being of nonclinical populations. Meta-analyses showed that MBIs have moderate effect size in reducing stress, psychological distress, depression, and anxiety among healthy individuals [19,20]. Internet-based MBIs are gaining more evidence as well. According to another review and meta-analysis, web-based MBIs are effective in reducing depressive symptoms, anxiety symptoms, stress, and improving well-being and mindfulness with small-to-medium effect size [21]. The above evidence provides the foundation to further develop internet-based MBIs for both clinical and healthy individuals.

### Mechanism of MBI

Regarding the mechanisms of MBIs in the promotion of well-being and the reduction of distress, the cultivation of mindfulness is found to mediate the relationship between MBIs and various outcome variables. For instance, Nyklíček and Kuijpers [22] found that changes in mindfulness partially mediated the relationship between mindfulness-based stress reduction intervention and its positive effects among people with distress symptoms. In addition to changes in mindfulness, according to Shapiro et al [23], the cultivation of mindfulness may also facilitate “reperceiving,” which is a shift of perspective that leads to an increased capacity for relating to one’s internal or external experiences objectively. For insomnia, mindfulness allows people to respond to stressors more skillfully by changing the patterns of worry and rumination that improve sleep quality [24]. For pain, preliminary evidence suggested that mindfulness and pain catastrophizing mediated the relationship between interventions and reduced perceived stress and improved quality of life [22,25]. For dysfunctional eating, MBI regulates appetitive and emotional processes by increasing both awareness and sensitivity to the eating process such that people can disengage themselves from the reactive eating habits [26]. To further test these mechanisms in this study, we hypothesized that text messaging–based MBIs would promote better well-being and fewer symptoms of insomnia, pain, and unhealthy eating through changes in both mindfulness and reperceiving.

### Text Messaging As the Core of Treatment Modality

Although internet-based MBIs were effective in reducing distress and promoting well-being [21], users’ retention and engagement remain challenging for digital health interventions [27,28]. A median of 56% retention was found, and the attrition rate can be as high as 75% for internet-based interventions [29,30]. Text messaging, considered as one of the future trends of internet-based interventions, may alleviate this problem, given the temporal synchronization feature of instant messaging that allows the intervention to catch people’s attention directly [31,32]. A systematic review showed that text messaging improves treatment adherence and reduces social isolation [33]. Up till now, to the best of our knowledge, text messaging is mostly used as a reminder, self-monitoring tool, or as an adjunct to face-to-face intervention instead of being utilized as the core treatment modality, except in a few physical health intervention studies [29,34-38]. Thus, the potential for text messaging to offer mental health intervention that improves users’ engagement and retention is yet to be investigated.

### Aim of This Study

The aim of this study was to test whether a text messaging–based MBI is effective in reducing distress related to insomnia, pain, and dysfunctional eating in 3 randomized controlled trials. WhatsApp [39] was chosen as the messaging tool because it is the most popular in the world with 2000 million monthly active users and is the most accessible option for the community [40]. Specifically, we aimed to investigate whether text messaging–based MBI can result in improvement in primary outcomes (ie, insomnia severity, pain severity, dysregulated eating) and secondary outcomes (ie, depressive and anxiety symptoms, mental well-being, and functional impairment). We also aimed to examine mindfulness, dysfunctional beliefs (for insomnia), pain catastrophizing (for chronic pain), and power of food (for dysregulated eating) as possible mechanisms of change that mediate the relationship between text messaging–based MBI with both primary and secondary outcomes.

## Methods

### Procedure

WhatsApp numbers for each trial were disseminated in the recruitment materials, and interested participants enrolled in the relevant trial via the instant messenger app, that is, WhatsApp. The nature and procedure of the studies were then explained to the participants through a WhatsApp message. Participants then completed the self-assessment web-based questionnaire together with a written consent page. Screening for eligibility of the participants was done upon completion of the preintervention questionnaire. Eligible participants were grouped into monthly batches, and the second author handled the enrollment, randomization, and intervention assignment of the participants. The randomization was conducted with Microsoft Office Excel 2010 [41] on an individual basis and 1:1 ratio between the intervention or wait-list control conditions with stratification on age (cutoff at 45 years old) and prior experience of mindfulness practice (no experience at all vs not novice). Participants were informed about their allocation of condition via WhatsApp by the same person who did the randomization. No blinding was feasible given the use of wait-list control in the design. A feasibility study on text messaging–based MBI had been done prior to this study. The intervention had a similar structure with this study. It was an uncontrolled study aiming at examining the impact of a general MBI, and the results revealed an improvement in the well-being and mindfulness of the participants. Therefore, the research team proceeded to these randomized controlled trial studies. Preintervention, postintervention, 1-month, and 3-month follow-up questionnaires were distributed via WhatsApp using Qualtrics [42]. Pretesting of the questionnaire before launch was done by the second author. The questionnaires were divided into 4 pages, namely, the consent form, demographic and screening items, primary outcomes, and secondary outcomes. The questionnaire pages were limited to around 50 items per page. Cookies were enabled, and participants could resume their questionnaire on the same device. Participants were required to input their research ID in the questionnaire, and duplicated data with the same research ID were eliminated. The postintervention questionnaires were sent to participants in both wait-list control and intervention conditions on the 22nd day. To adopt an intent-to-treat approach, all participants were invited to complete the self-report questionnaires at every time point regardless of whether they had completed the questionnaires at previous time points. Wait-list control participants received the intervention after completion of the 3-month follow-up questionnaire. Data were exported and stored as a password-protected Excel file in an encrypted flash drive.

### Ethics Approval

These studies were conducted in accordance with the ethical codes of the Declaration of Helsinki. Ethics approval was obtained from the Joint Chinese University of Hong Kong-New Territories East Cluster Clinical Research Ethics Committee (2017.203-T). Findings in this paper are reported in accordance with the recommendations of the Consolidated Standards of Reporting Trials (CONSORT).

### Participants

**Figure 1:**
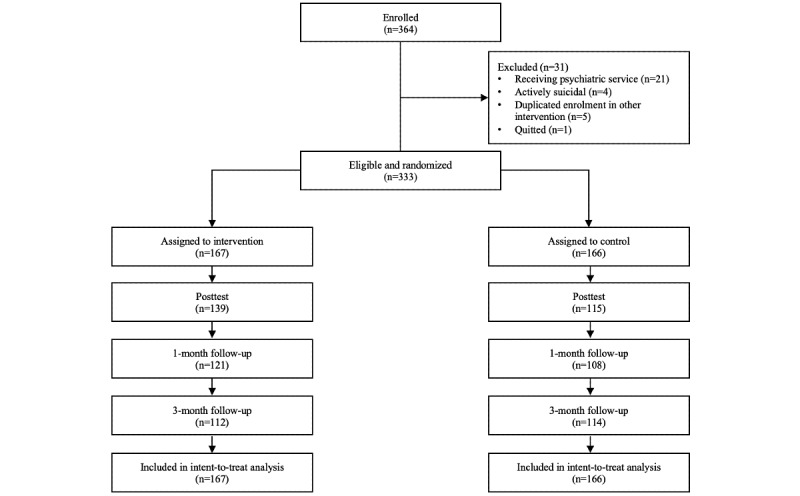
CONSORT diagram: enrollment and flowchart of study 1.

**Figure 2:**
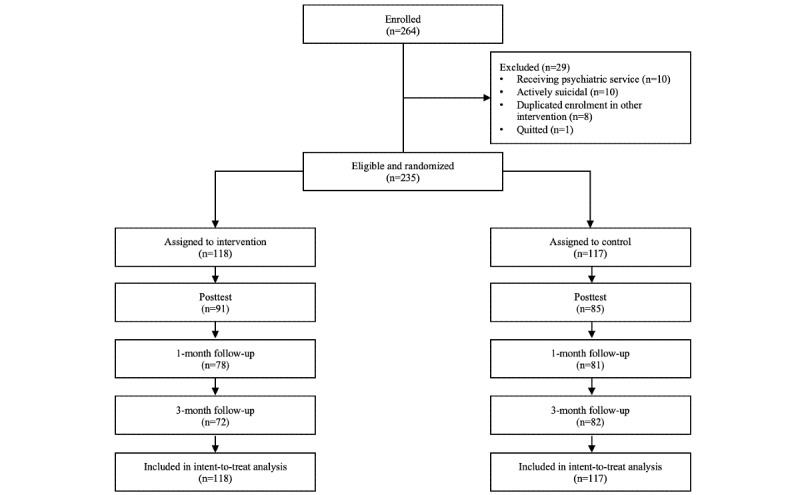
CONSORT diagram: enrollment and flowchart of study 2.

**Figure 3:**
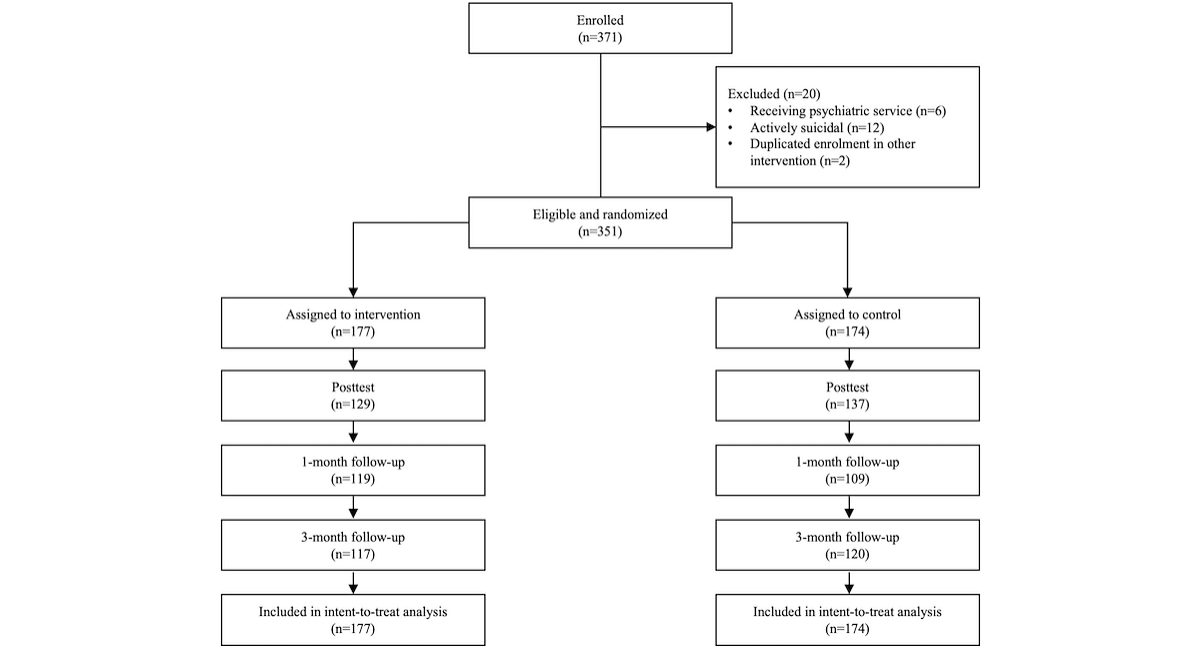
CONSORT diagram: enrollment and flowchart of study 3.

Participants were recruited from the general public via social networking platforms, a health-related information website, a local magazine, mass emails, and announcement postings at local tertiary institutions, collaborating nongovernmental organizations, and mutual-aid groups. Participants were quasi-anonymous. Multiple registrations to different trials were eliminated. Participants were only allowed to join one of the trials. The recruitment and follow-up lasted from July 2017 to October 2018; HKD 100 (US $12.87) was offered to 10 participants from each trial as an incentive via random draw. Eligibility criteria for participation included (1) age of 18 years or older, (2) ability to understand Cantonese and give consent, and (3) adequate level of computer literacy to follow the web-based instructions independently, together with daily access to the internet. Participants who self-reported receiving psychiatric services or active suicidality were excluded from this study; hotline and related resources were provided to those participants. No harm or other unintended effects were noticed in both conditions across all 3 trials. In the 3 trials, 364, 264, and 371 eligible participants were recruited for insomnia, pain, and dysregulated eating trials, respectively. Some of the participants withdrew from the studies or became out of reach after the assignment of condition. See Figure 1, Figure 2, and Figure 3 for the CONSORT diagrams of the 3 randomized controlled trials.

### Intervention

All MBIs were delivered in 3-week packages, with 21 days of daily mindfulness exercises that consist of an audio clip and a psychoeducational article delivered via WhatsApp. Participants were grouped into monthly batches, and they received the same messages on a fixed schedule. The intervention was technician-assisted when participants had any specific questions. One technician was involved, and the interaction was confined to confirmation of enrollment, explanation of the study procedures, general enquiry, and reminder for data collection as well as monitoring adherence. Standardized materials were delivered with broadcast function without other personalized messages. To enhance adherence, brief mindfulness exercises were used, which took around 10-15 minutes to complete. Participants were invited to indicate their adherence by replying on WhatsApp after finishing each exercise. Reminder messages were sent to those participants who had not completed any exercise within a week. See Multimedia Appendix 1 and Multimedia Appendix 2 for the outlines of the interventions. The delivery format referenced a WhatsApp-based intervention of Acceptance and Commitment Therapy with daily passage and audio clip [43]. Participants received text and audio from the research team through WhatsApp. Notifications were pushed. Progress could be tracked by checking if the participants had seen the message and listened to the audio in WhatsApp. See Multimedia Appendix 3 for the screenshots.

For insomnia and pain, the intervention content was designed by clinical psychologists referencing the mindfulness-based stress reduction program [44]. Initially, the intervention cultivates participants’ focused observation and awareness on bodily sensations, emotions, as well as thoughts. Practices included mindful breathing, mindful stretching, and body scan. Examples that are specific to insomnia and pain were included to increase relevance. Participants were encouraged to stay with aversive experiences with an open attitude. Lastly, suggestions were introduced to participants for integrating mindfulness practices in their daily life. For dysregulated eating, the MBI for eating incorporated 2 components running in parallel: (1) general mindfulness exercises and (2) specific eating-related components drawn from the Mindfulness-based Eating Awareness Training [26]. General mindfulness exercises started with observation and awareness of bodily sensations, emotions, and thoughts. Exercises on responding to unpleasant experiences were introduced. Meanwhile, eating-specific exercises were introduced to facilitate awareness of the eating experience. Specific exercises covered physical sensations of hunger and fullness, satisfaction of taste, changes in overall physical status, emotional triggers of eating, as well as choosing food with mindful awareness.

### Measurements

Three sets of questionnaires were designed for each trial. Primary outcomes were used only in specific trials that included the severity of the targeted health concern itself and the attitude toward the health concern. Secondary outcomes were used across 3 trials that covered functional impairment, emotional disturbance, and mental well-being, while mindfulness and specific reperceiving variables were measured as potential mediators. Participants were also invited to provide demographic information such as age, gender, religious belief, education level, employment status, personal income, and prior experience in meditation. Usage of medication to cope with sleep disturbance or pain was also measured. Use of mental health services was also recorded.

### Primary Outcomes of Study 1 on Insomnia

#### Insomnia Severity

Severity of insomnia was measured with the Insomnia Severity Index [45]. The Insomnia Severity Index consists of 7 items on a 5-point Likert scale. It is widely used as a screening tool and outcome measure [46-48]. The scale covers difficulties in falling asleep or maintaining sleep, early wakening, satisfaction with sleep quality, daytime functioning, and quality of life. The items constitute a single overall score, with higher scores indicating greater severity of the sleep problem. A locally validated translated Chinese version was used in this study [49]. The Chinese version of the Insomnia Severity Index demonstrated satisfactory internal consistency (Cronbach *α*=.83), 2-week test-retest reliability (*r*=0.79), and concurrent validity [50]. In this study, the scale achieved good internal consistency (Cronbach *α*=.80).

#### Presleep Arousal

The Presleep Arousal Scale [51] is a 16-item 5-point Likert scale that consists of 2 subscales measuring somatic and cognitive arousal when getting to sleep. Higher scores indicate higher arousal while getting to sleep. In this study, the scale was translated into Chinese with Brislin’s [52] forward and backward translation method. The 2 subscales of the Presleep Arousal Scale demonstrated satisfactory internal consistency in both the original validation study (Cronbach *α*=.76 and .81) [51] and this study (Cronbach *α*=.80 and .90).

#### Dysfunctional Beliefs and Attitude About Sleep (Reperceiving Mediator)

The Dysfunctional Beliefs and Attitude about Sleep Scale [53] is a 16-item scale measuring maladaptive beliefs and emotions about sleep difficulties. An 11-point Likert scale (0 “strongly disagree” to 10 “strongly agree”) is used, and higher scores indicate more dysfunctional thoughts and emotions. A validated 16-item abbreviated Taiwanese Chinese version of the Dysfunctional Beliefs and Attitude about Sleep [54] scale demonstrated satisfactory internal consistency in both the Taiwan validation study (Cronbach *α*=.87) and this study (Cronbach *α*=.82).

### Primary Outcomes of Study 2 on Pain

#### Pain Intensity

Subjective pain intensity was measured with a single-item Visual Analogue Scale (VAS). Participants were asked to indicate their overall pain intensity in the last week on a sliding scale from 0 to 100, with the anchor of “no pain,” “moderate pain,” and “extreme pain” on 0, 50, and 100, respectively. VAS is the most widely used tool for pain, and studies showed that VAS is valid and reliable tool that is sensitive in detection [55-57].

#### Pain Acceptance

The Chronic Pain Acceptance Questionnaire [58] was used to measure the willingness to accept pain and to maintain daily engagement despite the pain. This scale consists of 20 items on a 7-point Likert scale with 2 subscales: (1) activity engagement and (2) pain willingness. The overall score is based on the sum of the 2 subscale scores, with higher scores of Chronic Pain Acceptance Questionnaire indicating better acceptance of pain. The Chinese version of Chronic Pain Acceptance Questionnaire has been validated with good test-retest reliability (*r*=0.79), internal consistency (Cronbach *α*=.79), and concurrent validity [59]. In this study, the scale demonstrated good overall internal consistency (Cronbach *α*=.83).

#### Pain Catastrophizing (Reperceiving Mediator)

The Pain Catastrophizing Scale [60] was used to measure catastrophic beliefs and appraisals about pain. It consists of 13 items on a 5-point Likert scale, with higher scores indicating worse catastrophizing tendency toward pain. In this study, a locally validated Chinese version of the scale was used [61]. The scale demonstrated good internal consistency in both the validation study (Cronbach *α*=.93) and this study (Cronbach *α*=.94).

### Primary Outcomes of Study 3 on Dysregulated Eating

#### Eating Behaviors

Participants’ eating behaviors were measured by the 18-item Three-Factor Eating Questionnaire-Revised (TFEQ-R18) [62]. It consists of 18 four-point Likert scale items. The TFEQ-R18 consists of 3 subscales: (1) uncontrolled eating that refers to difficulties in regulating eating behaviors, (2) cognitive restraint that refers to conscious effort to inhibit food intake, and (3) emotional eating that refers to eating behaviors motivated by dysphoric mood, loneliness, or anxiety. In this study, the TFEQ-R18 has been translated into Chinese with Brislin’s [52] translation and back translation method. The scales demonstrated adequate-to-good internal consistency (Cronbach *α*=.76-.85) in the original validation study [62]. In this study, the scale of uncontrolled eating and emotional eating demonstrated good internal consistency (Cronbach *α*=.83-.88). However, the scale of cognitive restraint demonstrated acceptable internal consistency (Cronbach *α*=.68).

#### Reactivity to Food Cue (Reperceiving Mediator)

The Power of Food Scale [63] was used to measure food craving across contexts with different levels of proximity to food. It consists of 15 items on a 5-point Likert scale. Research results supported a single-factor model [64], and higher scores indicate stronger reactivity to food cue. The literature revealed satisfactory internal consistency of the scale (Cronbach *α*=.81-.91). In this study, the scale has been translated into Chinese using Brislin’s [52] forward and backward translation procedures. The translated scale demonstrated good internal consistency in this study (Cronbach *α*=.92).

### Secondary Outcomes in All Studies

#### Mindfulness (Mediator)

The Mindful Attention Awareness Scale [65] is a 15-item scale that measures trait mindfulness characterized by a present-oriented attention and awareness. All items are rated on a 6-point Likert scale, and higher scores indicate higher mindful awareness. The scale has been validated for use with healthy normal adults [65]. Research evidence supported a single-factor solution, and the scale demonstrated good internal consistency, test-retest reliability, discriminant, convergent validity, and criterion validity. It could also differentiate meditators from nonmeditators [65,66]. In this study, a Taiwanese Chinese version of the scale [67] was used that demonstrates good reliability in the Taiwan study (Cronbach *α*=.87) and this study (Cronbach *α*=.91).

#### Depression

The Patient Health Questionnaire [68] is a 9-item screening instrument for depressive symptoms. All items are rated on a 4-point Likert scale, and higher scores indicate more severe depressive symptoms. An officially translated Hong Kong traditional Chinese version was used in this study. Literature reported good internal consistency (Cronbach *α*=.86-.89) and criterion validity with 88% sensitivity and 88% specificity. In this study, the scale demonstrated good internal consistency (Cronbach *α*=.85).

#### Anxiety

The Generalized Anxiety Disorder scale [69] is a 7-item screening questionnaire for general anxiety symptoms. All items are rated on a 4-point Likert scale, with higher scores indicating more severe anxiety symptoms. An officially translated Hong Kong traditional Chinese version was used in this study. The scale demonstrated good internal consistency in both the original validation study (Cronbach *α*=.92) and this study (Cronbach *α*=.926). Literature reported good test-retest reliability (*r*=0.83) and criterion validity with 88% sensitivity and 89% specificity [69].

#### Mental Well-being

The World Health Organization Well-being Index [70] is a 5-item scale measuring mental well-being. All items are rated on a 6-point Likert scale and constitute a sum score. Higher scores indicate better mental well-being. An officially translated Chinese version was used in this study. Literature reported good internal consistency, concurrent validity, and sensitivity to change of the scale [71-73]. In this study, the scale demonstrated satisfactory internal consistency (Cronbach *α*=.91).

#### Functional Impairment

The Work and Social Adjustment Scale [74] measures functional impact due to health-related issues. The scale was used in the insomnia and pain studies. This scale consists of 5 items on different domains of functioning such as work, social, home, and leisure. All items were rated on a 9-point Likert scale, and higher scores indicate more severe impairment. Research evidence supported a single-factor solution of the scale and reported good internal consistency (Cronbach *α*=.93) [75]. A Chinese version of the scale was used in this study, which demonstrated good internal consistency (Cronbach *α*=.88).

### Analysis

Significance tests such as the independent 2-sided *t* tests and chi-square test of independence were employed to explore any baseline difference between completers and dropout participants. Intent-to-treat analyses were used in this study. Data of all the participants were included in the analysis regardless of their treatment adherence or attrition. Missing data were treated using multiple imputations, and 100 imputed data sets were generated. van Ginkel and Kroonenberg’s [76] method of repeated measures analysis of variance (ANOVA) using imputed data was used in this study. Specifically, missing values were estimated and 100 plausible complete versions of the data sets were created. Results from these 100 data sets were then pooled into 1 analysis by applying Rubin’s [77] pooling procedures. The pooling procedure was carried out using an SPSS macro by van Ginkel [78]. Treatment condition (intervention condition vs wait-list control) and time were entered as the fixed between-group and within-subject factors, respectively. To examine the mediating effects of mindfulness and reperceiving (ie, dysfunctional beliefs and attitudes about sleep, pain catastrophizing, power of food) on the relationship between condition and the primary and secondary outcomes, path analyses were conducted using Mplus 7 [79]. In the path analyses, condition was dummy coded (with the wait-list control condition coded as the reference group) and was treated as the independent variable. Mindfulness and condition-specific reperceiving mediator at postintervention assessment were treated as the mediators. Primary and secondary outcomes at 1-month and 3-month follow-up assessments were treated as the dependent variables. Baseline scores of all variables included in the model were controlled.

## Results

### Participant Characteristics

The demographic data and baseline characteristics of the participants are summarized in Table 1. Overall, the dropout rate was consistent between the intervention and wait-list control conditions (insomnia: *χ^2^*_1_ (N=333)=0.2; *P*=.62; pain: *χ^2^*_1_ (N=235)=1.2; *P*=.27; dysregulated eating: *χ^2^*_1_ (N=351)=0.1; *P*=.80). Chi-square tests and independent *t* tests revealed no significant association between the demographics variable and the dropout rate. No significant baseline difference between completers and dropout participants on any outcome measures was found.

**Table 1:** Demographic data and baseline characteristics of the participants.

Characteristics			Study 1: Insomnia (N=333)				Study 2: Pain (N=235)					Study 3: Dysregulated eating (N=351)		
			Intervention		Wait-list control		Intervention		Wait-list control		Intervention			Wait-list control
Age (years), mean (SD)			41.67 (13.57)		42.59 (13.08)		41.19 (15.45)		41.51 (13.66)		36.32 (11.64)			35.69 (11.55)
**Sex, n (%)**														
	Male	40 (24)		27 (16.2)		17 (14.4)		21 (18)		20 (11.3)			16 (9.2)	
	Female	126 (75.5)		139 (83.7)		101 (85.6)		96 (82.1)		157 (88.7)			158 (90.8)	
**Religion, n (%)**														
	Catholic	7 (4.2)		16 (9.6)		11 (9.3)		7 (6)		5 (2.8)			5 (2.9)	
	Christian	41 (24.6)		29 (17.5)		29 (24.6)		29 (24.8)		32 (18.1)			42 (24.1)	
	Buddhist	25 (15)		37 (22.3)		18 (15.3)		19 (16.2)		29 (16.4)			28 (16.1)	
	Others	1 (0.6)		1 (0.6)		0 (0)		0 (0)		1 (0.6)			2 (1.2)	
	None	92 (55.1)		83 (50)		60 (50.9)		62 (53)		110 (62.2)			97 (55.8)	
**Education, n (%)**														
	Primary	1 (0.6)		2 (1.2)		3 (2.5)		4 (3.4)		1 (0.6)			0 (0)	
	Junior secondary	7 (4.2)		6 (3.6)		4 (3.4)		8 (6.8)		2 (1.1)			0 (0)	
	Senior secondary	36 (21.6)		41 (24.7)		30 (25.4)		21 (18)		37 (20.9)			30 (17.2)	
	College/university	77 (46.1)		72 (43.4)		48 (40.7)		44 (37.6)		91 (51.4)			91 (52.3)	
	Master or above	43 (25.8)		43 (25.9)		31 (26.3)		40 (34.2)		46 (26)			53 (30.5)	
	Others	2 (1.2)		2 (1.2)		2 (1.7)		0 (0)		1 (0.6)			0 (0)	
**Employment, n (%)**														
	Student	18 (10.8)		15 (9)		7 (5.9)		10 (8.6)		31 (17.5)			30 (17.2)	
	Full-time	105 (62.9)		94 (56.6)		66 (55.9)		63 (53.9)		110 (62.2)			111 (63.8)	
	Part-time	8 (4.8)		9 (5.4)		10 (8.5)		11 (9.4)		13 (7.3)			13 (7.5)	
	Unemployed	3 (1.8)		1 (0.6)		4 (3.4)		3 (2.6)		0 (0)			4 (2.3)	
	Retired	19 (11.4)		25 (15.1)		19 (16.1)		14 (12)		5 (2.8)			7 (4)	
	Others	9 (5.4)		16 (9.6)		11 (9.3)		12 (10.3)		15 (8.5)			7 (4)	
**Previous experience in mindfulness practice, n (%)**														
	Yes	40 (24)		42 (25.3)		33 (28)		32 (27.4)		41 (23.2)			43 (24.7)	
	No	126 (75.5)		124 (74.7)		85 (72)		85 (72.6)		136 (76.8)			131 (75.3)	

### Study 1 on Insomnia

Correlations among the variables are presented in Table 2. Repeated measures ANOVA with the imputed data revealed significant intervention by time interaction effect on insomnia severity (*F*_3,790.1259_=7.434; *P*<.001), somatic presleep arousal (*F*_3,887.221_=4.504; *P*=.004), cognitive presleep arousal (*F*_3,795.662_=5.286; *P*=.001), dysfunctional beliefs and attitude about sleep (*F*_3,775.253_=5.784; *P*<.001), mindfulness (*F*_3,749.436_=3.590; *P*=.01), depression (*F*_3,838.002_=3.938; *P*=.008), anxiety (*F*_3,845.675_=4.554; *P*=.004), and mental well-being (*F*_3,826.162_=3.482; *P*=.02), whereas the intervention by time interaction effect on functional adjustment was found to be nonsignificant (*F*_3,681.529_=0.964; *P*=.41). Further analysis revealed that the intervention condition showed a better outcome on most of the outcome measures at postintervention, 1-month, and 3-month follow-up, compared with its respective wait-list control condition (see Table 3). Effect sizes are presented in Table 4.

**Table 2:** Correlations among variables of study 1 at baseline.

			ISI^a^		PSAS-S^b^		PSAS-C^c^		DBAS^d^		WSAS^e^		MAAS^f^		PHQ-9^g^		GAD-7^h^		WHO-5^i^
**Insomnia Severity Index**																			
	*r*	—^j^																	
	*P* value																		
**Presleep Arousal-Somatic**																			
	*r*	0.420		—															
	*P* value	<.001																	
**Presleep Arousal-Cognitive**																			
	*r*	0.489		0.605		—													
	*P* value	<.001		<.001															
**16-item Dysfunctional Beliefs and Attitudes about Sleep**																			
	*r*	0.401		0.330		0.387		—											
	*P*value	<.001		<.001		<.001													
**Work and Social Adjustment Scale**																			
	*r*	0.365		0.330		0.311		0.475		—									
	*P* value	<.001		<.001		<.001		<.001											
**Mindful Attention Awareness Scale**																			
	*r*	–0.237		–0.300		–0.405		–0.353		–0.335		—							
	*P* value	<.001		<.001		<.001		<.001		<.001									
**9-item Patient Health Questionnaire**																			
	*r*	0.555		0.451		0.479		0.441		0.461		–0.562		—					
	*P* value	<.001		<.001		<.001		<.001		<.001		<.001							
**7-item Generalized Anxiety Disorder Scale**																			
	*r*	0.461		0.483		0.590		0.449		0.436		–0.493		0.725		—			
	*P* value	<.001		<.001		<.001		<.001		<.001		<.001		<.001					
**5-item World Health Organization Well-being Index**																			
	*r*	–0.407		–0.250		–0.295		–0.263		–0.230		0.245		–0.517		–0.407		—	
	*P* value	<.001		<.001		<.001		<.001		<.001		<.001		<.001		<.001			

^a^ISI: Insomnia Severity Index.

^b^PSAS-S: Presleep Arousal-Somatic.

^c^PSAS-C: Presleep Arousal-Cognitve.

^d^DBAS: Dysfunctional Beliefs and Attitudes about Sleep.

^e^WSAS: Work and Social Adjustment Scale.

^f^MAAS: Mindful Attention Awareness Scale.

^g^PHQ-9: 9-item Patient Health Questionnaire.

^h^GAD-7: 7-item Generalized Anxiety Disorder Scale.

^i^WHO-5: 5-item World Health Organization Well-being Index.

^j^Not applicable.

**Table 3:** Repeated measures analysis of variance for study 1: intent-to-treat analysis (N=333).

			Preintervention		Postintervention		1-month follow-up		3-month follow-up		Interaction effect							Condition effect							Time effect				
			Mean		Mean		Mean		Mean		*F (df)*			*P* value			*F (df)*				*P* value			*F (df)*				*P* value	
**Insomnia Severity Index**												7.434 (3, 790.1259)			<.001				16.864 (1, 307.0944)			<.001				66.726 (3, 698.7932)			<.001
	Intervention	15.32		10.38		10.75		10.35																					
	Wait-list	15.40		12.88		12.97		12.82																					
**Presleep Arousal-Somatic**												4.504 (3, 887.221)			.004				6.544 (1, 312.570)			0.01				3.351 (3, 845.665)			.02
	Intervention	6.77		5.07		5.84		5.59																					
	Wait-list	6.73		6.64		6.79		7.59																					
**Presleep Arousal-Cognitive**												5.286 (3, 795.662)			.001				5.227 (1, 313.295)			.02				16.391 (3, 688.2804)			<.001
	Intervention	15.08		11.76		11.95		11.07																					
	Wait-list	14.89		13.89		13.40		13.79																					
**Dysfunctional Beliefs and Attitudes about Sleep**												5.784 (3, 775.253)			<.001				8.830 (1, 309.7971)			.003				9.690 (3, 646.1311)			<.001
	Intervention	76.58		69.95		69.22		68.86																					
	Wait-list	76.81		75.40		75.44		75.75																					
**Mindful Attention Awareness Scale**												3.590 (3, 749.436)			.01				1.072 (1, 318.1953)			.30				3.866 (3, 696.4202)			.009
	Intervention	4.06		4.25		4.31		4.29																					
	Wait-list	4.15		4.11		4.18		4.13																					
**9-item Patient Health Questionnaire**												3.938 (3, 838.0021)			.008				0.852 (1, 309.7324)			.36				11.400 (3, 785.1927)			<.001
	Intervention	9.56		7.57		7.35		7.60																					
	Wait-list	8.84		8.49		7.97		8.44																					
**7-item Generalized Anxiety Disorder Scale**												4.554 (3, 845.6747)			.004				2.132 (1, 316.0437)			.15				5.483 (3, 766.9396)			.001
	Intervention	8.61		6.78		6.82		6.99																					
	Wait-list	8.06		8.10		7.73		7.98																					
**5-item World Health Organization Well-being Index**												3.482 (3, 826.1617)			.02				3.221 (1, 304.5821)			.07				10.346 (3, 735.2119)			<.001
	Intervention	31.40		38.42		38.14		42.17																					
	Wait-list	32.84		33.69		34.82		36.29																					
**Work and Social Adjustment Scale**												0.964 (3, 681.5286)			.41				1.959 (1, 271.476)			.16				2.306 (3, 534.3949)			.08
	Intervention	17.83		16.07		15.19		16.02																					
	Wait-list	17.68		17.17		16.81		17.63																					

**Table 4:** Effect sizes of study 1.

			Postintervention versus preintervention									1-month follow-up versus preintervention									3-month follow-up versus preintervention						
			*t (df)*		*P* value		Cohen *d*		CI		*t (df)*			*P* value		Cohen *d*		CI		*t (df)*			*P* value		Cohen *d*		CI
**Insomnia Severity Index**																											
	Intervention	13.143 (5443.323)		<.001		1.11		0.92 to 1.31		10.392 (2254.874)			<.001		0.97		0.78 to 1.16		11.762 (1547.873)			<.001		1.04		0.85 to 1.23	
	Wait-list	7.038 (1622.922)		<.001		1.06		0.78 to 1.34		5.606 (894.225)			<.001		0.50		0.34 to 0.65		6.416 (1233.278)			<.001		0.53		0.38 to 0.67	
**Presleep Arousal-Somatic**																											
	Intervention	4.451 (15081.001)		<.001		0.34		0.19 to 0.49		2.184 (6574.37)			.03		0.19		0.03 to 0.34		2.637 (4766.154)			.008		0.23		0.07 to 0.40	
	Wait-list	0.267 (2637.896)		.79		0.04		–0.21 to 0.28		–0.143 (3473.035)			.89		0.01		–0.16 to 0.14		–1.977 (2875.841)			.048		–0.16		–0.03 to –0.01	
**Presleep Arousal-Cognitive**																											
	Intervention	6.794 (8818.592)		<.001		0.49		0.35 to 0.64		5.234 (1371.045)			<.001		0.45		0.30 to 0.61		6.884 (1663.291)			<.001		0.58		0.43 to 0.74	
	Wait-list	2.086 (1186.225)		.04		0.28		0.05 to 0.50		2.797 (1085.731)			.005		0.21		0.08 to 0.33		2.092 (2344.107)			.04		0.15		0.02 to 0.27	
**16-item Dysfunctional Beliefs and Attitudes about Sleep**																											
	Intervention	6.397 (3434.157)		<.001		0.43		0.30 to 0.56		5.976 (1548.938)			<.001		0.45		0.31 to 0.59		5.662 (1459.862)			<.001		0.45		0.31 to 0.59	
	Wait-list	1.349 (1526.281)		.18		0.18		0.05 to 0.42		1.143 (564.757)			.25		0.09		–0.03 to 0.21		0.863 (873.599)			.39		–0.07		–0.06 to 0.19	
**Mindful Attention Awareness Scale**																											
	Intervention	–3.659 (3932.992)		<.001		0.23		0.12 to 0.35		–4.724 (1464.818)			<.001		0.31		0.20 to 0.43		–3.830 (1361.077)			<.001		0.28		0.15 to 0.40	
	Wait-list	0.668 (1370.984)		.50		–0.07		–0.27 to 0.13		–0.670 (975.659)			.50		–0.05		–0.15 to 0.05		0.207 (1054.694)			.84		–0.01		–0.13 to 0.10	
**9-item Patient Health Questionnaire**																											
	Intervention	5.456 (5123.518)		<.001		0.43		0.28 to 0.58		5.864 (2734.949)			<.001		0.48		0.33 to 0.64		5.003 (2211.678)			<.001		0.41		0.26 to 0.56	
	Wait-list	1.014 (2969.454)		.31		0.14		–0.11 to 0.39		2.361 (1450.462)			.02		0.18		0.05 to 0.31		1.049 (2210.411)			.29		0.08		–0.05 to 0.22	
**7-item Generalized Anxiety Disorder Scale**																											
	Intervention	5.022 (8639.716)		<.001		0.38		0.23 to 0.53		4.685 (2436.844)			<.001		0.37		0.23 to 0.52		4.308 (2432.34)			<.001		0.33		0.19 to 0.47	
	Wait-list	–0.102 (2277.929)		.92		–0.02		–0.26 to 0.23		0.832 (1421.884)			.41		0.07		–0.07 to 0.20		0.225 (2365.867)			.82		0.02		–0.11 to 0.15	
**5-item World Health Organization Well-being Index**																											
	Intervention	–4.387 (2744.74)		<.001		0.36		0.21 to 0.51		–4.417 (2159.869)			<.001		0.35		0.21 to 0.49		–6.318 (1335.566)			<.001		0.55		0.39 to 0.71	
	Wait-list	–0.523 (2592.251)		.60		–0.09		–0.40 to 0.22		–1.303 (1338.226)			.19		–0.11		–0.24 to 0.03		–2.088 (1592.958)			.04		–0.19		–0.34 to –0.03	
**Work and Social Adjustment Scale**																											
	Intervention	2.107 (822.965)		.04		0.21		0.05 to 0.37		3.233 (743.299)			.001		0.31		0.16 to 0.47		2.257 (778.492)			.02		0.21		0.06 to 0.37	
	Wait-list	0.601 (674.646)		.55		0.12		–0.20 to 0.45		0.970 (394.396)			.33		0.1		–0.05 to 0.26		0.063 (688.404)			.95		0		–0.15 to 0.16	

### Mediation Model

#### One-Month Follow-up Results

The results showed that all 4 models had satisfactory model fit. In addition, at 1-month follow-up assessment, the indirect effects of condition on all primary and secondary outcomes through dysfunctional beliefs and attitudes about sleep were significant, except mental well-being. The indirect effects of condition through mindfulness were significant on cognitive presleep arousal.

#### Three-Month Follow-up Results

**Figure 4:**
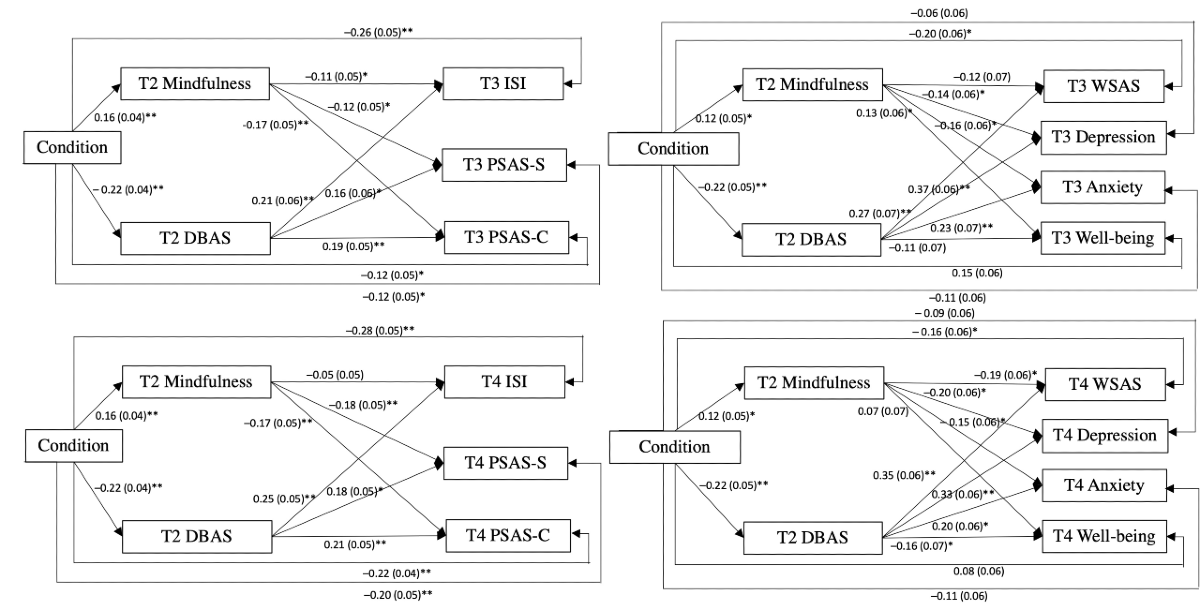
Path models of study 1. Baseline variables were controlled. For the purpose of clarity, the controlled variables and the covariance of the residuals between variables are not depicted. DBAS: Dysfunctional Beliefs and Attitudes about Sleep; ISI: Insomnia Severity Index; PSAS-C: Presleep Arousal-Cognitive; PSAS-S: Presleep Arousal-Somatic; T2: postassessment; T3: 1-month follow-up assessment; T4: 3-month follow-up assessment; WSAS: Work and Social Adjustment Scale. *P<.05, **P<.001.

At 3-month follow-up assessment, all 4 models showed satisfactory model fit. The indirect effects of condition on the primary and secondary outcomes through dysfunctional beliefs and attitudes about sleep were all significant. However, the indirect effects of condition through mindfulness were only significant on cognitive and somatic presleep arousal. Table 5 shows a summary of the model fit, standardized path coefficients, standard errors, indirect effects, total effects, and model fits of the 4 models. Figure 4 shows the mediation models of study 1.

**Table 5:** Standardized path coefficients, standard errors, indirect effects, total effects, and model fits of the mediation analyses in study 1.

Model, dependent variables		a (Condition → Mindfulness)	b (Condition → Dysfunctional Beliefs and Attitude about Sleep Scale)	c (Mindfulness → dependent variable^c^)	d (Dysfunctional Beliefs and Attitude about Sleep Scale → dependent variable)	e (Condition → dependent variable)	Indirect effect through mindfulness (a^*^c)	Indirect effect through Dysfunctional Beliefs and Attitude about Sleep Scale (b^*^d)	Total effect
**1a, Primary outcomes at 1-month follow-up^a^**									
	Insomnia Severity Index	0.155 (0.042), *P*<.001	–0.224 (0.041), *P*<.001	–0.109 (0.053), *P*=.04	0.212 (0.058), *P*<.001	–0.262 (0.051), *P*<.001	–0.017 (0.010), *P*=.08	–0.047 (0.015), *P*=.002	–0.327 (0.049), *P*<.001
	Presleep Arousal-Somatic	N/A^b^	N/A	–0.119 (0.052), *P*=.02	0.164 (0.055), *P*=.003	–0.124 (0.051), *P*=.02	–0.018 (0.010), *P*=.05	–0.037 (0.014), *P*=.008	–0.179 (0.050), *P*<.001
	Presleep Arousal-Cognitive	N/A	N/A	–0.169 (0.048), *P*<.001	0.194 (0.051), *P*<.001	–0.116 (0.047), *P*=.01	–0.026 (0.010), *P*=.01	–0.043 (0.014), *P*=.001	–0.186 (0.047), *P*<.001
**1b, Primary outcomes at 3-month follow-up^c^**									
	Insomnia Severity Index	0.155 (0.042), *P*<.001	–0.224 (0.041), *P*<.001	–0.050 (0.051), *P*=.33	0.249 (0.053), *P*<.001	–0.281 (0.048), *P*<.001	–0.008 (0.008), *P*=.35	–0.056 (0.016), *P*<.001	–0.344 (0.047), *P*<.001
	Presleep Arousal-Somatic	N/A	N/A	–0.175 (0.050), *P*<.001	0.180 (0.054) *P*=.001	–0.201 (0.048), *P*<.001	–0.027 (0.011), *P*=.01	–0.040 (0.014), *P*=.004	–0.268 (0.046), *P*<.001
	Presleep Arousal-Cognitive	N/A	N/A	–0.164 (0.047), *P*<.001	0.210 (0.049), *P*<.001	–0.217 (0.044), *P*<.001	–0.025 (0.010), *P*=.01	–0.047 (0.014), *P*=.001	–0.289 (0.043), *P*<.001
**2a, Secondary outcomes at 1-month follow-up^d^**									
	Work and Social Adjustment Scale	0.119 (0.050), *P*=.02	–0.216 (0.048), *P*<.001	–0.120 (0.066), *P*=.07	0.272 (0.066), *P*<.001	–0.203 (0.060), *P*=.001	–0.014 (0.010), *P*=.16	–0.059 (0.019), *P*=.002	–0.277 (0.060), *P*<.001
	9-item Patient Health Questionnaire	N/A	N/A	–0.0140 (0.058), *P*=.02	0.370 (0.058), *P*<.001	–0.059 (0.059), *P*=.32	–0.017 (0.010), *P*=.09	–0.080 (0.022), *P*<.001	–0.156 (0.061), *P*=.01
	7-item Generalized Anxiety Disorder Scale	N/A	N/A	–0.160 (0.064), *P*=.01	0.229 (0.065), *P*<.001	–0.106 (0.061), *P*=.08	–0.019 (0.011), *P*=.09	–0.049 (0.018), *P*=.005	–0.174 (0.060), *P*=.004
	5-item World Health Organization Well-being Index	N/A	N/A	0.133 (0.063), *P*=.03	–0.113 (0.068), *P*=.09	0.154 (0.060), *P*=.01	0.016 (0.010), *P*=.12	0.024 (0.016), *P*=.12	0.194 (0.059), *P*=.001
**2b, Secondary outcomes at 3-month follow-up^e^**									
	Work and Social Adjustment Scale	0.119 (0.050), *P*=.02	–0.216 (0.048), *P*<.001	–0.191 (0.060), *P*=.001	0.346 (0.064), *P*<.001	–0.157 (0.059), *P*=.008	–0.023 (0.012), *P*=.06	–0.075 (0.021), *P*<.001	–0.254 (0.060), *P*<.001
	9-item Patient Health Questionnaire	N/A	N/A	–0.196 (0.061), *P*=.001	0.327 (0.061), *P*<.001	–0.088 (0.059), *P*=.13	–0.023 (0.012), *P*=.06	–0.071 (0.021), *P*=.001	–0.182 (0.059), *P*=.002
	7-item Generalized Anxiety Disorder Scale	N/A	N/A	–0.148 (0.064), *P*=.02	0.201 (0.064), *P*=.002	–0.110 (0.059), *P*=.06	–0.018 (0.011), *P*=.11	–0.043 (0.017), *P*=.009	–0.171 (0.059), *P*=.004
	5-item World Health Organization Well-being Index	N/A	N/A	0.071 (0.071), *P*=.32	–0.158 (0.071), *P*=.03	0.084 (0.063), *P*=.18	0.008 (0.009), *P*=.37	0.034 (0.017), *P*=.048	0.127 (0.061), *P*=.04

^a^Model fit: *χ^2^*_20_=31.3; *P*=.051; comparative fit index=0.988; Tucker–Lewis index=0.976; standardized root mean squared residual=0.029; root mean square error of approximation=0.041

^b^N/A: not applicable.

^c^Model fit: *χ^2^*_20_=39.8; *P*=.005; comparative fit index=0.981; Tucker–Lewis index=0.961; standardized root mean squared residual=0.033; root mean square error of approximation=0.055.

^d^Model fit: *χ^2^*_30_=53.2; *P*=.006; comparative fit index=0.970; Tucker–Lewis index=0.942; standardized root mean squared residual=0.044; root mean square error of approximation=0.057.

^e^Model fit: *χ^2^*_30_=40.5; *P*=.09; comparative fit index=0.985; Tucker–Lewis index=0.971; standardized root mean squared residual=0.032; root mean square error of approximation=0.038.

### Study 2 on Pain

Correlations among variables are presented in Table 6. Repeated measures ANOVA results showed significant intervention condition by time interaction effect on pain acceptance (*F*_3,535.514_=4.186; *P*=.006), and pain catastrophizing (*F*_3,558.550_=3.179; *P*=.02), mindfulness (*F*_3,597.884_=3.003; *P*=.03), depression (*F*_3,592.642_=2.781; *P*=.04), and mental well-being (*F*_3,593.052_=3.762; *P*=.01). Meanwhile, interaction effects were found to be nonsignificant on subjective intensity of pain (*F*_3,591.397_=0.464; *P*=.71), functional adjustment (*F*_3,541.571_=1.720; *P*=.16), and anxiety (*F*_3,569.171_=2.230; *P*=.08). Further analysis showed that the significant interaction effects were largely maintained across postintervention, 1-month, and 3-month follow-ups. Participants in the intervention condition reported better outcomes on most of the measures (See Table 7). Effect sizes are presented in Table 8.

**Table 6:** Correlations among variables of study 2 at baseline.

		VAS^a^	CPAQ^b^	PCS^c^	WSAS^d^	MAAS^e^	PHQ-9^f^	GAD-7^g^	WHO-5^h^
**Visual Analogue Scale**									
	*r*	—^i^							
	*P* value								
**Chronic Pain Acceptance Questionnaire**									
	*r*	–0.405	—						
	*P* value	<.001							
**Pain Catastrophizing Scale**									
	*r*	0.449	–0.642	—					
	*P* value	<.001	<.001						
**Work and Social Adjustment Scale**									
	*r*	0.438	–0.603	0.635	—				
	*P* value	<.001	<.001	<.001					
**Mindful Attention Awareness Scale**									
	*r*	–0.117	0.276	–0.418	–0.344	—			
	*P* value	0.07	<.001	<.001	<.001				
**9-item Patient Health Questionnaire**									
	*r*	0.214	–0.325	0.483	0.371	–0.627	—		
	*P* value	<.001	<.001	<.001	<.001	<.001			
**7-item Generalized Anxiety Disorder Scale**									
	*r*	0.174	–0.327	0.470	0.434	–0.554	0.751	—	
	*P* value	0.007	<.001	<.001	<.001	<.001	<.001		
**5-item World Health Organization Well-being Index**									
	*r*	–0.218	0.391	–0.466	–0.342	0.426	–0.613	–0.598	—
	*P* value	<.001	<.001	<.001	<.001	<.001	<.001	<.001	

^a^VAS: Visual Analogue Scale.

^b^CPAQ: Chronic Pain Acceptance Questionnaire.

^c^PCS: Pain Catastrophizing Scale.

^d^WSAS: Work and Social Adjustment Scale.

^e^MAAS: Mindful Attention Awareness Scale.

^f^PHQ-9: 9-item Patient Health Questionnaire.

^g^GAD-7: 7-item Generalized Anxiety Disorder Scale.

^h^WHO-5: 5-item World Health Organization Well-being Index.

^i^Not applicable.

**Table 7:** Repeated measures analysis of variance for study 2: intent-to-treat analysis.

			Preinterventionn		Postintervention		1-month follow-up		3-month follow-up		Interaction effect							Condition effect							Time effect				
			Mean		Mean		Mean		Mean		*F (df)*			*P* value			*F (df)*				*P* value			*F (df)*				*P* value	
**Visual Analogue Scale**												0.464 (3, 591.3966)			.71				6.525 (1, 210.2334)			.01				9.533 (3, 552.7345)			<.001
	Intervention	49.36		40.09		39.28		40.91																					
	Wait-list	52.74		45.56		46.09		47.92																					
**Chronic Pain Acceptance Questionnaire**												4.186 (3, 535.5144)			.006				1.015 (1, 216.9613)			.32				6.591 (3, 508.6055)			<.001
	Intervention	58.96		64.41		64.49		63.85																					
	Wait-list	60.88		63.16		60.57		61.18																					
**Pain Catastrophizing Scale**												3.179 (3, 558.5502)			.02				3.370 (1, 215.56)			.07				5.365 (3, 535.191)			.001
	Intervention	2.84		2.52		2.56		2.53																					
	Wait-list	2.81		2.75		2.75		2.79																					
**Mindful Attention Awareness Scale**												3.003 (3, 597.8836)			.03				5.068 (1, 220.251)			.03				1.958 (3, 511.7978)			.12
	Intervention	4.19		4.37		4.39		4.47																					
	Wait-list	4.15		4.12		4.19		4.11																					
**9-item Patient Health Questionnaire**												2.781 (3, 592.6418)			.04				0.062			.80				1.445 (3, 533.4394)			.23
	Intervention	7.60		6.50		6.95		10.88																					
	Wait-list	7.77		8.25		8.39		12.75																					
**7-item Generalized Anxiety Disorder Scale**												2.230 (3, 569.1707)			.08				6.244 (1, 218.5466)			.01				0.777 (3, 559.0639)			.51
	Intervention	6.80		5.82		6.33		6.30																					
	Wait-list	7.17		7.66		8.08		7.96																					
**5-item World Health Organization Well-being Index**												3.762 (3, 593.0518)			.01				1.886 (1, 216.6309)			.17				2.281 (3, 578.2624)			.08
	Intervention	39.93		48.11		46.50		45.79																					
	Wait-list	41.85		39.55		41.83		43.92																					
**Work and Social Adjustment Scale**												1.720 3, 541.5711)			.16				0.535 (1, 200.4921)			.47				4.790 (3, 437.0823)			.003
	Intervention	17.79		13.42		15.32		13.88																					
	Wait-list	16.40		14.84		16.64		15.43																					

**Table 8:** Effect sizes of study 2.

		Postintervention versus preintervention					1-month follow-up versus preintervention					3-month follow-up versus preintervention			
		*t (df)*	*P* value	Cohen *d*	CI	*t (df)*		*P* value	Cohen *d*	CI	*t (df)*		*P* value	Cohen *d*	CI
**Visual Analogue Scale**															
	Intervention	4.236 (3020.777)	<.001	0.47	0.26 to 0.67	4.042 (2266.412)		<.001	0.48	0.27 to 0.71	3.230 (1716.028)		.001	0.40	0.18 to 0.62
	Wait-list	3.351 (1895.399)	.001	0.35	0.16 to 0.54	2.929 (1427.677)		.003	0.32	0.13 to 0.50	2.04 (1866.139)		.04	0.23	0.03 to 0.42
**Chronic Pain Acceptance Questionnaire**															
	Intervention	–5.597 (3163.368)	<.001	0.45	0.30 to 0.61	–4.763 (1184.051)		<.001	0.45	0.28 to 0.62	–3.467 (822.627)		.001	0.38	0.57 to 0.57
	Wait-list	–2.187 (1276.259)	.03	0.18	0.04 to 0.32	0.297 (1075.033)		.77	–0.03	–0.16 to 0.11	–0.260 (992.647)		.79	0.02	–0.12 to 0.17
**Pain Catastrophizing Scale**															
	Intervention	4.801 (2844.541)	<.001	0.42	0.26 to 0.59	3.949 (1366.464)		<.001	0.38	0.21 to 0.55	3.616 (1387.779)		<.001	0.38	0.20 to 0.57
	Wait-list	0.920 (1543.763)	.36	0.08	–0.07 to 0.22	0.872 (1408.471)		.38	0.08	–0.06 to 0.22	0.286 (1009.163)		.78	0.03	–0.12 to 0.18
**Mindful Attention Awareness Scale**															
	Intervention	–2.593 (4982.628)	.01	0.24	0.07 to 0.41	–2.726 (2285.433)		.006	0.27	0.09 to 0.45	–3.255 (1058.504)		.001	0.35	0.17 to 0.54
	Wait-list	0.391 (1549.393)	.69	–0.04	–0.17 to 0.10	–0.579 (1657.309)		.56	0.05	–0.09 to 0.18	0.490 (1088.182)		.62	–0.04	–0.18 to 0.09
**9-item Patient Health Questionnaire**															
	Intervention	2.832 (3259.39)	.005	0.25	0.09 to 0.42	1.504 (1163.214)		.13	0.14	0.02 to 0.29	0.880 (799.257)		.38	0.09	–0.07 to 0.25
	Wait-list	–1.261 (2701.169)	.21	–0.09	–0.21 to 0.04	–1.360 (1804.568)		.17	–0.12	–0.26 to 0.03	–2.615 (1727.604)		.009	–0.23	–0.38 to **–**0.07
**7-item Generalized Anxiety Disorder Scale**															
	Intervention	2.537 (1765.07)	.01	0.24	0.08 to 0.41	1.088 (717.43)		.28	0.11	0.05 to 0.27	1.118 (1134.925)		.26	0.11	0.06 to 0.29
	Wait-list	–1.267 (2817.28)	.21	–0.09	–0.22 to 0.04	–2.057 (1693.877)		.04	–0.17	–0.31 to **–**0.03	–1.634 (2480.039)		.10	–0.14	–0.29 to 0.01
**5-item World Health Organization Well-being Index**															
	Intervention	–4.207 (4037.397)	<.001	0.38	0.21 to 0.56	–3.530 (1700.367)		<.001	0.31	0.15 to 0.46	–2.231 (1714.783)		.03	0.27	0.06 to 0.48
	Wait-list	1.169 (1678.009)	.24	–0.11	–0.27 to 0.05	0.007 (1799.488)		.99	0	–0.16 to 0.16	–1.028 (1669.093)		.30	0.09	0.06 to 0.25
**Work and Social Adjustment Scale**															
	Intervention	4.328 (690.693)	<.001	0.49	0.30 to 0.68	2.241 (614.852)		.03	0.27	0.08 to 0.45	3.377 (712.65)		.001	0.43	0.22 to 0.64
	Wait-list	1.569 (671.049)	.12	0.17	0.00 to 0.34	–0.213 (834.573)		.83	–0.03	–0.22 to 0.16	0.935 (688.875)		.35	0.11	–0.07 to 0.29

### Mediation Model

#### One-Month Follow-up Assessment

Results showed that condition had significant indirect effects on pain intensity and functional adjustment through pain catastrophizing, whereas condition showed significant indirect effects on depression and anxiety through mindfulness. All models showed satisfactory model fit.

#### Three-Month Follow-up Assessment

**Figure 5:**
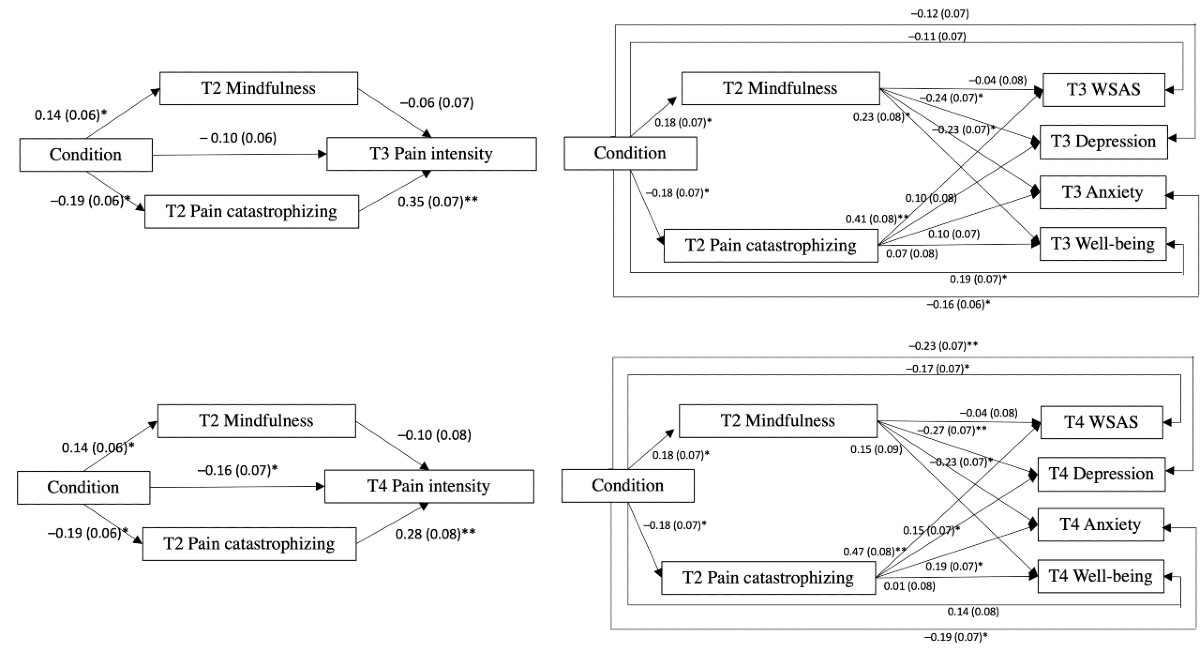
Path models of study 2. Baseline variables were controlled. For the purpose of clarity, the controlled variables and the covariance of the residuals between variables are not depicted. T2: postassessment; T3: 1-month follow-up assessment; T4: 3-month follow-up assessment; WSAS: Work and Social Adjustment Scale. *P<.05, **P<.001.

Similar to results at 1-month follow-up, the indirect effects of condition through pain catastrophizing on pain intensity and functional adjustment at 3-month follow-up were significant, and the indirect effects of condition through mindfulness on depression and anxiety were also significant. A summary of the model fit and standardized coefficients are shown in Table 9 and Figure 5.

**Table 9:** Standardized path coefficients, standard errors, indirect effects, total effects, and model fit of the mediation analyses in study 2.

Dependent variables		a (Condition → Mindfulness)	b (Condition → Pain Catastrophizing Scale)	c (Mindfulness → dependent variable)	d (Pain Catastrophizing Scale → dependent variable)	e (Condition → dependent variable)	Indirect effect through mindfulness (a*c)	Indirect effect through Pain Catastrophizing Scale (b*d)	Total effect
**Primary outcomes at 1-month follow-up^a^**									
	Pain intensity	0.141 (0.056), *P*=.01	–0.188 (0.057), *P*=.001	–0.061 (0.071), *P*=.39	0.350 (0.072), *P*<.001	–0.096 (0.063), *P*=.13	–0.009 (0.011), *P*=.42	–0.066 (0.025), *P*=.007	–0.171 (0.064), *P*=.007
**Primary outcomes at 3-month follow-up^b^**									
	Pain intensity	0.141 (0.056), *P*=.01	–0.188 (0.057), *P*=.001	–0.099 (0.076), *P*=.19	0.281 (0.077), *P*<.001	–0.164 (0.065), *P*=.01	–0.014 (0.012), *P*=.26	–0.053 (0.022), *P*=.02	–0.231 (0.064), *P*<.001
**Secondary outcomes at 1-month follow-up^c^**									
	Work and Social Adjustment Scale	0.178 (0.065), *P*=.006	–0.181 (0.065), *P*=.005	–0.042 (0.078), *P*=.59	0.408 (0.083), *P*<.001	–0.107 (0.074), *P*=.15	–0.008 (0.015), *P*=.61	–0.074 (0.032), *P*=.02	–0.189 (0.074), *P*=.01
	9-item Patient Health Questionnaire	N/A^d^	N/A	–0.244 (0.074), *P*=.001	0.103 (0.075), *P*=.17	–0.121 (0.065), *P*=.06	–0.044 (0.021), *P*=.04	–0.019 (0.016), *P*=.23	–0.184 (0.065), *P*=.005
	7-item Generalized Anxiety Disorder Scale	N/A	N/A	–0.226 (0.071), *P*=.002	0.099 (0.070), *P*=.16	–0.161 (0.064), *P*=.012	–0.041 (0.020), *P*=.045	–0.018 (0.015), *P*=.22	–0.219 (0.063), *P*=.001
	5-item World Health Organization Well-being Index	N/A	N/A	0.228 (0.084), *P*=.007	0.068 (0.084), *P*=.42	0.185 (0.070), *P*=.008	0.041 (0.022), *P*=.06	–0.012 (0.016), *P*=.45	0.214 (0.068), *P*=.002
**Secondary outcomes at 3-month follow-up^e^**									
	Work and Social Adjustment Scale	0.178 (0.065) *P*=.006	–0.181 (0.065), *P*=.005	–0.042 (0.080), *P*=.59	0.472 (0.082), *P*<.001	–0.169 (0.074), *P*=.02	–0.008 (0.015), *P*=.61	–0.086 (0.035), *P*=.02	–0.263 (0.073), *P*<.001
	9-item Patient Health Questionnaire	N/A	N/A	–0.265 (0.073), *P*<.001	0.149 (0.071), *P*=.04	–0.232 (0.065), *P*<.001	–0.047 (0.022), *P*=.03	–0.027 (0.017), *P*=.10	–0.306 (0.064), *P*<.001
	7-item Generalized Anxiety Disorder Scale	N/A	N/A	–0.232 (0.074), *P*=.002	0.192 (0.071), *P*=.007	–0.192 (0.066), *P*=.004	–0.042 (0.021), *P*=.04	–0.035 (0.019), *P*=.06	–0.268 (0.066), *P*<.001
	5-item World Health Organization Well-being Index	N/A	N/A	0.152 (0.085), *P*=.07	0.007 (0.084), *P*=.93	0.140 (0.076), *P*=.07	0.027 (0.018), *P*=.14	–0.001 (0.016), *P*=.93	0.165 (0.074), *P*=.03

^a^Model fit: *χ^2^*_6_=5.5; *P*=.49; comparative fit index=1.00; Tucker–Lewis index=1.005; standardized root mean squared residual=0.034; root mean square error of approximation<.001

^b^Model fit: *χ^2^*_6_=6.2; **P*=*.40; comparative fit index=0.999; Tucker–Lewis index=0.998; standardized root mean squared residual=0.34; root mean square error of approximation=0.011.

^c^Model fit: *χ^2^*_30_=29.2; *P*=.51; comparative fit index=1.00; Tucker–Lewis index=1.003; standardized root mean squared residual=0.04; root mean square error of approximation<.001.

^d^N/A: not applicable.

^e^Model fit: *χ^2^*_30_=25.9; *P*=.68; comparative fit index=1.00; Tucker–Lewis index=1.013; standardized root mean squared residual=0.040; root mean square error of approximation<.001.

### Study 3 on Dysregulated Eating

Correlations among variables are presented in Table 10. Results of repeated measures ANOVA showed significant intervention condition by time interaction effect on uncontrolled eating (*F*_3,912.364_=3.041; *P*=.03), emotional eating (*F*_3,944.138_=4.294; *P*=.005), mindfulness (*F*_3,854.310_=2.670; *P*=.05), and mental well-being (*F*_3,882.777_=4.457; *P*=.004). Meanwhile, analyses on other measures showed nonsignificant results. For the outcome with significant results, further analyses showed that significant group difference was maintained and the intervention condition reported better outcomes at postintervention, 1-month, and 3-month follow-up (See Table 11). Effect sizes are presented in Table 12.

**Table 10:** Correlations among variables of study 3 at baseline.

		TFEQ-UE^a^	TFEQ-CR^b^	TFEQ-EE^c^	PFS^d^	MAAS^e^	PHQ-9^f^	GAD-7^g^	WHO-5^h^
**Three-Factor Eating Questionnaire-Uncontrolled Eating**									
	*r*	—^i^							
	*P* value								
**Three-Factor Eating Questionnaire-Cognitive Restraint**									
	*r*	–0.038	—						
	*P* value	.48							
**Three-Factor Eating Questionnaire-Emotional Eating**									
	*r*	0.643	–0.009	—					
	*P* value	<.001	.87						
**Power of Food Scale**									
	*r*	0.708	–0.048	0.538	—				
	*P* value	<.001	.37	<.001					
**Mindful Attention Awareness Scale**									
	*r*	–0.368	0.110	–0.423	–0.358	—			
	*P* value	<.001	.04	<.001	<.001				
**9-item Patient Health Questionnaire**									
	*r*	0.251	–0.116	0.373	0.288	–0.626	—		
	*P* value	<.001	.03	<.001	<.001	<.001			
**7-item Generalized Anxiety Disorder Scale**									
	*r*	0.200	–0.027	0.304	0.206	–0.564	0.796	—	
	*P* value	<.001	.62	<.001	<.001	<.001	<.001		
**5-item World Health Organization Well-being Index**									
	*r*	–0.086	0.107	–0.243	–0.102	0.46	–0.592	–0.578	—
	*P* value	0.11	.05	<.001	.06	<.001	<.001	<.001	

^a^TFEQ-UE: Three-Factor Eating Questionnaire-Uncontrolled Eating.

^b^TFEQ-CR: Three-Factor Eating Questionnaire-Cognitive Restraint.

^c^TFEQ-EE: Three-Factor Eating Questionnaire-Emotional Eating.

^d^PFS: Power of Food Scale.

^e^MAAS: Mindful Attention Awareness Scale.

^f^PHQ-9: 9-item Patient Health Questionnaire.

^g^GAD-7: 7-item Generalized Anxiety Disorder Scale.

^h^WHO-5: 5-item World Health Organization Well-being Index.

^i^Not applicable.

**Table 11:** Repeated measures analysis of variance for study 3: intent-to-treat analysis (N=351).

			Preintervention		Postintervention		1-month follow-up		3-month follow-up		Interaction effect						Condition effect							Time effect			
			Mean		Mean		Mean		Mean		*F (df)*			*P* value			*F (df)*			*P* value			*F (df)*			*P* value	
**Three-Factor Eating Questionnaire-Uncontrolled Eating**												3.041 (3, 912.364)			.03			10.275 (1, 325.2121)			.002			21.132 (3, 841.623)			<.001
	Intervention	20.95		19.21		18.97		18.70																			
	Wait-list	21.53		21.06		20.40		20.37																			
**Three-Factor Eating Questionnaire-Cognitive Restraint**												0.135 (3, 875.081)			.94			0.086 (1, 303.5693)			.77			3.299 (3, 793.3058)			.02
	Intervention	9.21		9.51		9.59		9.50																			
	Wait-list	9.12		9.52		9.52		9.35																			
**Three-Factor Eating Questionnaire-Emotional Eating**												4.294 (3, 944.138)			.005			7.781 (1, 324.9344)			.006			4.792 (3, 866.233)			.003
	Intervention	7.58		6.86		6.88		6.84																			
	Wait-list	7.61		7.58		7.52		7.57																			
**Power of Food Scale**												0.771 (3, 1031.464)			.51			19.932 (1, 343.1272)			<.001			23.943 (3, 1030.6991)			<.001
	Intervention	43.64		40.38		40.32		39.16																			
	Wait-list	47.22		45.89		44.56		43.98																			
**Mindful Attention Awareness Scale**												2.670 (3, 854.310)			.05			5.346 (1, 310.4349)			.02			7.599 (3, 793.6769)			<.001
	Intervention	4.05		4.29		4.31		4.33																			
	Wait-list	4.00		4.05		4.08		4.16																			
**9-item Patient Health Questionnaire**												1.241 (3, 914.466)			.29			2.715 (1, 320.5567)			.10			4.236 (3, 826.3964)			.006
	Intervention	7.61		6.72		6.27		6.37																			
	Wait-list	7.74		7.75		6.99		7.19																			
**7-item Generalized Anxiety Disorder Scale**												1.75 (3, 900.082)			.16			1.557 (1, 323.1843)			.21			2.048 (3, 825.7671)			.11
	Intervention	6.99		5.87		5.93		5.89																			
	Wait-list	6.86		6.89		6.50		6.57																			
**5-item World Health Organization Well-being Index**												4.457 (3, 882.777)			.004			1.890 (1, 320.3858)			.17			2.178 (3, 789.4446)			.09
	Intervention	42.97		50.12		48.36		48.68																			
	Wait-list	44.94		43.92		46.67		44.78																			

**Table 12:** Effect sizes of study 3.

				Postintervention versus Preintervention									1-month follow-up versus Preintervention									3-month follow-up versus Preintervention						
				*t (df)*		*P* value		Cohen *d*		CI		*t (df)*			*P* value		Cohen *d*		CI		*t (df)*			*P* value		Cohen *d*		CI
**Three-Factor Eating Questionnaire-Uncontrolled Eating**																												
	Intervention		5.186 (3817.101)		<.001		0.36		0.23 to 0.50		6.105 (3639.106)			<.001		0.45		0.31 to 0.59		5.861 (2319.303)			<.001		0.46		0.32 to 0.61	
	Wait-list		1.930 (4614.144)		.05		0.12		0.01 to 0.23		2.893 (3664.02)			.004		0.20		0.07 to 0.33		3.170 (2504.465)			.002		0.24		0.12 to 0.37	
**Three-Factor Eating Questionnaire-Cognitive Restraint**																												
	Intervention		–1.643 (2879.543)		.005		0.13		–0.01 to 0.28		–1.801 (1687.066)			.07		0.16		0.01 to 0.31		–1.366 (1069.425)			.17		0.12		–0.03 to 0.27	
	Wait-list		–2.440 (3497.992)		.02		0.18		0.05 to 0.31		–2.106 (2073.137)			.04		0.17		0.03 to 0.30		–1.303 (2226.666)			.19		0.10		–0.03 to 0.23	
**Three-Factor Eating Questionnaire-Emotional Eating**																												
	Intervention		4.721 (1990.064)		.001		0.33		0.20 to 0.45		4.393 (2491.901)			<.001		0.32		0.19 to 0.46		4.431 (3790.093)			<.001		0.35		0.20 to 0.49	
	Wait-list		0.197 (3848.245)		.84		0.01		–0.11 to 0.14		0.111 (2995.514)			.91		0.00		–0.13 to 0.14		0.320 (2534.89)			.75		0.02		–0.11 to 0.15	
**Power of Food Scale**																												
		Intervention		4.381 (50588.352)		<.001		0.30		0.16 to 0.43		4.367 (121149.766)			<.001		0.34		0.18 to 0.49		4.769 (113062.726)			<.001		0.46		0.32 to 0.61
		Wait-list		2.739 (112506.843)		.006		0.17		0.05 to 0.30		3.570 (27839.166)			<.001		0.24		0.11 to 0.36		5.298 (62137.205)			<.001		0.35		0.22 to 0.48
**Mindful Attention Awareness Scale**																												
	Intervention		–3.686 (1565.495)		.001		0.27		0.14 to 0.39		–4.124 (1323.734)			<.001		0.29		0.17 to 0.41		–4.180 (1425.676)			<.001		0.12		–0.03 to 0.27	
	Wait-list		–0.940 (5467.915)		.35		0.07		–0.07 to 0.20		–0.524 (2393.459)			.60		0.03		–0.09 to 0.16		–1.551 (2324.842)			.12		0.12		–0.02 to 0.26	
**9-item Patient Health Questionnaire**																												
	Intervention		2.321 (3106.031)		.12		0.17		0.04 to 0.30		2.948 (3308.172)			.003		0.25		0.10 to 0.40		2.868 (2623.881)			.004		0.35		0.20 to 0.49	
	Wait-list		–0.099 (6576.707)		.92		0.01		–0.15 to 0.14		1.502 (2597.217)			.13		0.12		–0.02 to 0.26		0.815 (2368.508)			.42		0.07		0.08 to 0.21	
**7-item Generalized Anxiety Disorder Scale**																												
	Intervention		3.096 (2600.162)		.047		0.21		0.09 to 0.34		2.373 (2117.166)			.02		0.18		0.05 to 0.32		2.646 (2359.088)			.008		0.39		0.23 to 0.55	
	Wait-list		–0.171 (6545.757)		.86		–0.01		–0.15 to 0.13		0.344 (4111.079)			.73		0.03		–0.12 to 0.17		0.347 (2103.419)			.73		0.03		–0.12 to 0.18	
**5-item World Health Organization Well-being Index**																												
	Intervention		–4.243 (3063.119)		.02		0.33		0.19 to 0.48		–2.920 (1302.715)			.004		0.22		0.09 to 0.36		–2.651 (1620.939)			.008		0.33		0.19 to 0.46	
	Wait-list		0.885 (4256.998)		.38		–0.07		–0.21 to 0.07		–0.487 (1512.967)			.63		0.04		–0.10 to 0.17		0.628 (2220.244)			.53		0.06		–0.21 to 0.10	

### Mediation Model

#### One-Month Follow-up Results

Results showed that at 1-month follow-up assessments, condition showed significant indirect effects on uncontrolled eating and emotional eating through power of food. Condition also showed indirect effects on depression and anxiety through mindfulness.

#### Three-Month Follow-up Results

**Figure 6:**
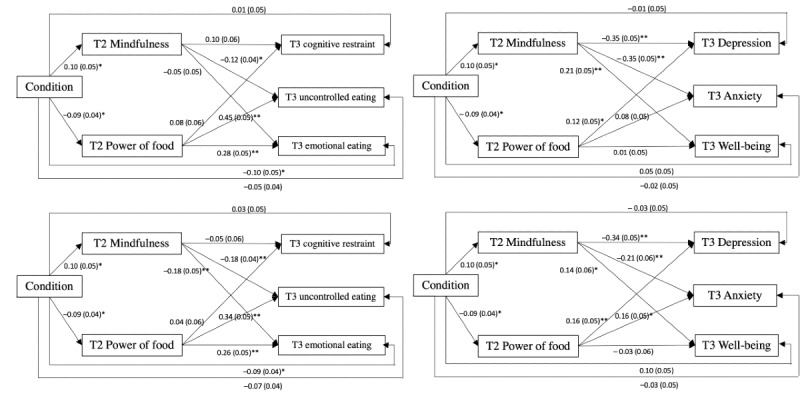
Path models of study 3. Baseline variables were controlled. For the purpose of clarity, the controlled variables and the covariance of the residuals between variables are not depicted. T2: postassessment; T3: 1-month follow-up assessment; T4: 3-month follow-up assessment. *P<.05, **P<.001.

At 3-month follow-up assessments, condition showed significant indirect effects on uncontrolled eating and emotional eating through power of food, whereas it showed significant indirect effect on depression through mindfulness. A summary of the model fit, standardized coefficients, indirect effects, and total effects is shown in Table 13 and Figure 6.

**Table 13:** Standardized path coefficients, indirect effects, total effects, and model fit of the mediation analyses in study 3.

Dependent variables		a (Condition → Mindfulness)	b (Condition → Power of Food Scale)	c (Mindfulness → dependent variable)	d (Power of Food Scale → dependent variable)	e (Condition → dependent variable)	Indirect effect through mindfulness (a^*^c)	Indirect effect through Power of Food Scale (b^*^d)	Total effect
**Primary outcomes at 1-month follow-up^a^**									
	Three-Factor Eating Questionnaire-Cognitive Restraint	0.100 (0.046), *P*=.03	–0.093 (0.037), *P*=.01	0.096 (0.055), *P*=.08	0.083 (0.056), *P*=.14	0.006 (0.053), *P*=.90	0.010 (0.007), *P*=.19	–0.008 (0.006), *P*=.21	0.008 (0.051), *P*=.87
	Three-Factor Eating Questionnaire-Uncontrolled Eating	N/A^b^	N/A	–0.120 (0.042), *P*=.005	0.447 (0.048), *P*<.001	–0.048 (0.039), *P*=.22	–0.012 (0.007), *P*=.09	–0.042 (0.017), *P*=.02	–0.101 (0.042), *P*=.02
	Three-Factor Eating Questionnaire-Emotional Eating	N/A	N/A	–0.049 (0.051), *P*=.33	0.275 (0.054), *P*<.001	–0.103 (0.046), *P*=.03	–0.005 (0.006), *P*=.40	–0.026 (0.011), *P*=.02	–0.134 (0.046), *P*=.004
**Primary outcomes at 3-month follow-up^c^**									
	Three-Factor Eating Questionnaire-Cognitive Restraint	0.100 (0.046), *P*=.03	–0.093 (0.037), *P*=.01	–0.054 (0.055), *P*=.33	0.037 (0.060), *P*=.54	0.029 (0.050), *P*=.57	–0.005 (0.006), *P*=.39	–0.003 (0.006), *P*=.57	0.020 (0.049), *P*=.69
	Three-Factor Eating Questionnaire-Uncontrolled Eating	N/A	N/A	–0.176 (0.044), *P*<.001	0.340 (0.053), *P*<.001	–0.071 (0.040), *P*=.08	–0.017 (0.009), *P*=.06	–0.032 (0.013), *P*=.02	–0.121 (0.043), *P*=.005
	Three-Factor Eating Questionnaire-Emotional Eating	N/A	N/A	–0.180 (0.046), *P*<.001	0.261 (0.048), *P*<.001	–0.093 (0.041), *P*=.02	–0.018 (0.009), *P*=.06	–0.024 (0.011), *P*=.02	–0.136 (0.043), *P*=.001
**Secondary outcomes at 1-month follow-up^d^**									
	9-item Patient Health Questionnaire	0.100 (0.046), *P*=.03	–0.093 (0.037), *P*=.01	–0.349 (0.053), *P*<.001	0.121 (0.054), *P*=.03	–0.006 (0.048), *P*=.90	–0.035 (0.017), *P*=.04	–0.011 (0.007), *P*=.09	–0.052 (0.049), *P*=.29
	7-item Generalized Anxiety Disorder Scale	N/A	N/A	–0.351 (0.052), *P*<.001	0.080 (0.052), *P*=.12	–0.020 (0.046), *P*=.66	–0.035 (0.017), *P*=.04	–0.007 (0.006), *P*=.19	–0.063 (0.048), *P*=.19
	5-item World Health Organization Well-being Index	N/A	N/A	0.206 (0.054), *P*<.001	0.009 (0.052), *P*=.87	0.048 (0.048), *P*=.31	0.021 (0.011), *P*=.07	–0.001 (0.005), *P*=.87	0.068 (0.047), *P*=.15
**Secondary outcomes at 3-month follow-up^e^**									
	9-item Patient Health Questionnaire	0.100 (0.046), *P*=.03	–0.093 (0.037), *P*=.01	–0.337 (0.053), *P*<.001	0.155 (0.051), *P*=.003	–0.028 (0.047), *P*=.545	–0.034 (0.016), *P*=.04	–0.014 (0.008), *P*=.06	–0.077 (0.049), *P*=.12
	7-item Generalized Anxiety Disorder Scale	N/A	N/A	–0.211 (0.055), *P*<.001	0.157 (0.054), *P*=.004	–0.031 (0.049), *P*=.52	–0.021 (0.011), *P*=.06	–0.015 (0.008), *P*=.06	–0.067 (0.049), *P*=.18
	5-item World Health Organization Well-being Index	N/A	N/A	0.141 (0.060), *P*=.02	–0.034 (0.056), *P*=.54	0.097 (0.052), *P*=.06	0.014 (0.009), *P*=.11	0.003 (0.006), *P*=.56	0.114 (0.051), *P*=.03

^a^Model fit: *χ^2^*_20_=37.8; *P*=.009; comparative fit index=0.981; Tucker–Lewis index=0.962; standardized root mean squared residual=0.029; root mean square error of approximation=0.050.

^b^N/A: not applicable.

^c^Model fit: *χ^2^*_20_=32.5; *P*=.04; comparative fit index=0.987; Tucker–Lewis index=0.973; standardized root mean squared residual=0.027; root mean square error of approximation=0.042.

^d^Model fit: *χ^2^*_20_=44.2; *P*=.002; comparative fit index=0.977; Tucker–Lewis index=0.954; standardized root mean squared residual=0.041; root mean square error of approximation=0.06.

^e^Model fit: *χ^2^*_20_=28.6; *P*=.09; comparative fit index=0.991; Tucker–Lewis index=0.983; standardized root mean squared residual=0.035; root mean square error of approximation=0.035.

## Discussion

### Principal Results

This study hypothesized that text messaging–based MBI is effective in reducing distress related to insomnia, pain, and dysfunctional eating. It was encouraging that effectiveness was demonstrated in all 3 randomized controlled trials. Most outcomes had improved significantly at 1-month follow-up compared to wait-list control condition and some effects were able to sustain at 3-month follow-up, including both primary outcomes (eg, insomnia, pain, dysregulated eating indicators) and secondary outcomes (eg, depressive, anxiety symptoms). Our findings showed that text messaging–based interventions not only serve as an adjunct or supplementary tool for traditional treatment programs but also can serve as the core treatment modality. Not only the effectiveness is promising, the retention rates are also high across 3 trials at postmeasurement for intervention groups, ranging from 72.9% (129/177) up to 83.2% (139/167), which demonstrated that text messaging–based intervention is a feasible solution that can maintain a high retention rate. This study contributed to the knowledge of how we can utilize the text messaging application to a fuller extent for intervention purposes so that people with a range of health-related issues can consider evidence-based options delivered via mobile messaging technology.

### Study 1 on Insomnia

The results of the randomized controlled trials demonstrated the effectiveness of the text messaging–based MBI on insomnia. Primary outcomes, namely, insomnia intensity, dysfunctional beliefs and attitudes about sleep, and presleep arousal were found to be significantly reduced compared to those in the wait-list control condition, and the effect was sustained at 3-month follow-up. Significant improvement of secondary outcomes, including depressive symptoms, anxiety symptoms, and mental well-being, were also found with sustained effect at 3-month follow-up. The results were consistent with those reported in previous studies on MBIs for insomnia that MBIs are effective in improving insomnia, depression, and anxiety and increasing mindfulness [12,13].

Dysfunctional beliefs and attitudes about sleep mediated the effect of the intervention on all primary outcomes and most secondary outcomes at both 1-month and 3-month follow-up. Mindfulness was found to mediate the effect of the intervention on presleep arousal (cognitive) at 1-month follow-up with additional mediating effects on presleep arousal (somatic) at 3-month follow-up. The mediating effects of dysfunctional beliefs and attitudes about sleep on the outcomes aligned with the mechanism proposed that it was suggested that mindfulness can improve sleep by changing the pattern of worry and rumination [24]. In this study, the mediation model showed that improved sleep might be made possible through reduced dysfunctional beliefs about sleep. However, it was unclear why mindfulness has a mediation effect only on the primary outcomes but not on the secondary outcomes. This pattern might be explained by the specificity of content that was targeted at insomnia.

### Study 2 on Pain

The results of repeated measures ANOVA showed that MBIs are effective in enhancing chronic pain acceptance and reducing pain catastrophizing at 3-month follow-up in the intervention group compared to those in the wait-list control group. Mindfulness, depressive symptoms, and mental well-being were also found to improve significantly at 3-month follow-up. However, no significant improvement was found in pain intensity, which can be explained by the non–symptom-focused approach of acceptance as it aims to improve pain adjustment independent of pain intensity [58]. Other results were largely consistent with previous research that MBI improved pain acceptance, pain catastrophizing, mindfulness, depression, and well-being [14-16]. However, anxiety was not reduced significantly, which might be explained by the floor effect that the baseline score was low. For the mediation model, preliminary evidence suggested that pain catastrophizing mediated the effect of MBI on pain intensity and functioning at both 1 month and 3 months, whereas mindfulness was only found to be mediating the effect of intervention on secondary outcomes but not primary outcomes. Our findings were consistent with a previous study conducted by Elvery et al [25] that pain catastrophizing emerged as the most robust process and it was the most predictive of pain intensity compared to pain acceptance and mindfulness. However, other studies showed a different process and mechanism wherein pain catastrophizing did not mediate the outcome but pain acceptance did, which might imply that the psychological process may be more important than cognitive process [80]. Further research is needed to verify the mechanism of how MBI leads to change in various outcomes.

### Study 3 on Dysregulated Eating

MBI was found to be effective in improving uncontrolled eating, emotional eating, and well-being at 3-month follow-up compared to the wait-list control condition. Nonsignificant results were found in other primary and secondary outcomes. However, for data without 3-month follow-up, a significant interaction effect was found for mindfulness. Owing to the complexity of eating issues, the materials used in this condition may not be focused enough to induce significant and sustained change. Preliminary moderation analysis showed that participants with higher uncontrolled eating scores showed greater reduction in uncontrolled eating at postintervention in this study. This finding implies that the intervention may have greater benefits for people who have more severe uncontrolled eating problems. The results of the mediation analysis showed that the power of food mediated the effect of intervention on both uncontrolled and emotional eating at both 1-month and 3-month follow-ups. Mindfulness was also found to mediate the effect on depressive symptoms at both 1-month and 3-month follow-ups. The pattern of mediation is similar to that in the pain condition that mindfulness only mediates secondary outcomes but not primary outcomes. Given little research has been conducted on the relationship between MBI and eating concerns, further research is needed to confirm the model.

### Implications

This study took a significant step in demonstrating the effectiveness of using text messaging–based MBIs to alleviate distress related to insomnia, pain, and dysregulated eating, which were affecting at least a quarter of the general population. Such interventions are potentially scalable to the population level and can be widely disseminated with relatively low costs and human resources. This novel way of delivering MBIs showed a high retention rate ranging from 72.9% (129/177) to 83.2% (139/167) for intervention groups at postintervention, which is higher than the median retention rate of internet-based interventions [29]. Given the potency of the text messaging MBIs in alleviating specific health concerns and general psychological distress, text messaging–based intervention deserves more attention in the future in delivering other forms of psychological interventions. Not only as adjunct to traditional face-to-face psychological interventions, text-messaging interventions can potentially become a core intervention modality that has high user engagement and positive treatment outcomes. This study also shed light on the mediation models of MBIs, which have not been thoroughly investigated. Across the 3 randomized controlled trials, mindfulness was found to mediate the effect of intervention on both primary and secondary outcomes. Condition-specific mediators were also found, including dysfunctional beliefs and attitudes about sleep for insomnia, pain catastrophizing for pain, and power of food for dysregulated eating. These findings supported that the cultivation of mindfulness and the ability to reperceive the present experience can alleviate health-related concerns and distress. Future designs for MBIs can consider focusing on these mechanisms of change. Nonetheless, replication is needed to confirm the mediation processes and how different mechanisms are related to different outcomes.

### Limitations

This study had several limitations that warrant attention. First, wait-list control was used instead of active control that can account for demand characteristics. As this study focused on investigating the effects of text messaging–based interventions among urban dwellers, comparison with wait-list controls is closer to the real-life situation where people generally do not seek help for their health-related conditions. Even though the placebo effect cannot be ruled out, as the active control group was not included [81], these findings demonstrated effectiveness in a wide range of psychological outcomes for 3 common health concerns.

Second, this study solely adopted self-report measures that may lack objectivity on the severity of health concerns such as sleep habits, pain severity, and eating patterns. Given the interventions were delivered over WhatsApp, the use of self-report measures that are completed online is consistent with accessing the intervention materials over mobile text messaging. Nevertheless, future studies may consider including behavioral or physiological measures to corroborate with self-report findings.

Third, the preponderance of the participants were women. The results of this study may not be generalizable to other genders. The skewness is consistent with gender difference in help-seeking, where men are less likely to seek help than women [82] and women are more interested in practicing mindfulness [83]. A systematic review also found that among 117 studies of randomized controlled trials of mindfulness-based cognitive therapy or mindfulness-based stress reduction with 9820 participants, only 29% of the total participants were men [84]. Future studies on smartphone-based MBIs should sample more men to examine whether these interventions may be more acceptable to men and conducive to their well-being.

Lastly, despite the high retention rates at postmeasurement, the return rates of questionnaires were low at 3-month follow-up, ranging from 61% (72/118) to 67.1% (112/167) for intervention groups and 68.7% (114/166) to 70.1% (82/117) for wait-list control groups. In future studies, strategies are needed to boost the return rates of questionnaires so that the sustained effectiveness can be captured more accurately. One of the possible solutions may be increasing the incentive for completing questionnaires at long follow-up periods.

### Conclusions

To conclude, this study showed that text messaging–delivered MBIs are effective in improving issues related to sleep, pain, and eating. Text messaging has the potential to be a core intervention modality to cater to the needs of people with a fast-paced lifestyle or increase accessibility to MBIs. The demonstrated mechanisms of change illuminate directions for future design of materials and focus on MBIs. Given the increasing health needs of the general population and low availability of evidence-based face-to-face interventions, text messaging–based interventions provide a viable alternative to expand on the availability of effective interventions to the public.

## Appendix

Multimedia Appendix 1 Outline of the intervention for study 1 and study 2.

Multimedia Appendix 2 Outline of the intervention for study 3.

Multimedia Appendix 3 Screenshots of the intervention (translated mock-up).

Multimedia Appendix 4 CONSORT-eHEALTH checklist (V 1.6.1).

## Abbreviations

ANOVA: analysis of variance
CONSORT: Consolidated Standards of Reporting Trials
MBI: mindfulness-based intervention
TFEQ-R18: 18-item Three-Factor Eating Questionnaire-Revised
VAS: visual analogue scale

## References

[1] Lam LC, Wong CS, Wang M, Chan W, Chen EY, Ng RM, Hung S, Cheung EF, Sham P, Chiu HF, Lam M, Chang W, Lee EH, Chiang T, Lau JT, van Os J, Lewis G, Bebbington P (2015). Prevalence, psychosocial correlates and service utilization of depressive and anxiety disorders in Hong Kong: the Hong Kong Mental Morbidity Survey (HKMMS). Soc Psychiatry Psychiatr Epidemiol.

[2] Morin CM, Rodrigue S, Ivers H (2003). Role of stress, arousal, and coping skills in primary insomnia. Psychosom Med.

[3] Davis MC, Zautra AJ, Smith BW (2004). Chronic pain, stress, and the dynamics of affective differentiation. J Pers.

[4] Tryon M, DeCant R, Laugero K (2013). Having your cake and eating it too: A habit of comfort food may link chronic social stress exposure and acute stress-induced cortisol hyporesponsiveness. Physiology & Behavior.

[5] Hafner M, Stepanek M, Taylor J, Troxel WM, van Stolk C (2017). Why sleep matters—the economic costs of insufficient sleep: a cross-country comparative analysis. Rand Health Q.

[6] Maniadakis N, Gray A (2000). The economic burden of back pain in the UK. Pain.

[7] Scarborough P, Bhatnagar P, Wickramasinghe KK, Allender S, Foster C, Rayner M (2011). The economic burden of ill health due to diet, physical inactivity, smoking, alcohol and obesity in the UK: an update to 2006-07 NHS costs. J Public Health (Oxf).

[8] Wong W, Fielding R (2011). Prevalence of insomnia among Chinese adults in Hong Kong: a population-based study. J Sleep Res.

[9] Wong WS, Fielding R (2011). Prevalence and characteristics of chronic pain in the general population of Hong Kong. J Pain.

[10] Centre for health protection, department of health: fruit consumption. Hong Kong Special Administrative Region.

[11] Kabat-Zinn J (2001). Mindfulness Meditation for Everyday Life.

[12] Zhang J, Liu X, Xie X, Zhao D, Shan M, Zhang X, Kong X, Cui H (2015). Mindfulness-based stress reduction for chronic insomnia in adults older than 75 years: a randomized, controlled, single-blind clinical trial. Explore (NY).

[13] Klatt M, Norre C, Reader B, Yodice L, White S (2016). Mindfulness in Motion: a Mindfulness-Based Intervention to Reduce Stress and Enhance Quality of Sleep in Scandinavian Employees. Mindfulness.

[14] Lauche R, Cramer H, Dobos G, Langhorst J, Schmidt S (2013). A systematic review and meta-analysis of mindfulness-based stress reduction for the fibromyalgia syndrome. J Psychosom Res.

[15] Veehof MM, Oskam M, Schreurs KMG, Bohlmeijer ET (2011). Acceptance-based interventions for the treatment of chronic pain: a systematic review and meta-analysis. Pain.

[16] Anheyer Dennis, Haller Heidemarie, Barth Jürgen, Lauche Romy, Dobos Gustav, Cramer Holger (2017). Mindfulness-Based Stress Reduction for Treating Low Back Pain: A Systematic Review and Meta-analysis. Ann Intern Med.

[17] Godfrey KM, Gallo LC, Afari N (2015). Mindfulness-based interventions for binge eating: a systematic review and meta-analysis. J Behav Med.

[18] O'Reilly GA, Cook L, Spruijt-Metz D, Black DS (2014). Mindfulness-based interventions for obesity-related eating behaviours: a literature review. Obes Rev.

[19] Galante J, Friedrich C, Dawson AF, Modrego-Alarcón Marta, Gebbing P, Delgado-Suárez Irene, Gupta R, Dean L, Dalgleish T, White IR, Jones PB (2021). Mindfulness-based programmes for mental health promotion in adults in nonclinical settings: A systematic review and meta-analysis of randomised controlled trials. PLoS Med.

[20] Querstret D, Morison L, Dickinson S, Cropley M, John M (2020). Mindfulness-based stress reduction and mindfulness-based cognitive therapy for psychological health and well-being in nonclinical samples: A systematic review and meta-analysis. International Journal of Stress Management.

[21] Spijkerman MPJ, Pots WTM, Bohlmeijer ET (2016). Effectiveness of online mindfulness-based interventions in improving mental health: A review and meta-analysis of randomised controlled trials. Clin Psychol Rev.

[22] Nyklícek Ivan, Kuijpers KF (2008). Effects of mindfulness-based stress reduction intervention on psychological well-being and quality of life: is increased mindfulness indeed the mechanism?. Ann Behav Med.

[23] Shapiro SL, Carlson LE, Astin JA, Freedman B (2006). Mechanisms of mindfulness. J Clin Psychol.

[24] Lundh L (2005). The Role of Acceptance and Mindfulness in the Treatment of Insomnia. J Cogn Psychother.

[25] Elvery N, Jensen M, Ehde D, Day M (2017). Pain catastrophizing, mindfulness, and pain acceptance: what?s the difference?. Clin J Pain.

[26] Kristeller J, Wolever RQ, Sheets V (2013). Mindfulness-Based Eating Awareness Training (MB-EAT) for Binge Eating: A Randomized Clinical Trial. Mindfulness.

[27] Alkhaldi G, Hamilton FL, Lau R, Webster R, Michie S, Murray E (2016). The Effectiveness of Prompts to Promote Engagement With Digital Interventions: A Systematic Review. J Med Internet Res.

[28] Torous J, Nicholas J, Larsen ME, Firth J, Christensen H (2018). Clinical review of user engagement with mental health smartphone apps: evidence, theory and improvements. Evid Based Ment Health.

[29] Waller R, Gilbody S (2009). Barriers to the uptake of computerized cognitive behavioural therapy: a systematic review of the quantitative and qualitative evidence. Psychol Med.

[30] Kaltenthaler E, Sutcliffe P, Parry G, Beverley C, Rees A, Ferriter M (2008). The acceptability to patients of computerized cognitive behaviour therapy for depression: a systematic review. Psychol Med.

[31] Barak A, Grohol JM (2011). Current and Future Trends in Internet-Supported Mental Health Interventions. Journal of Technology in Human Services.

[32] Free C, Phillips G, Galli L, Watson L, Felix L, Edwards P, Patel V, Haines A (2013). The effectiveness of mobile-health technology-based health behaviour change or disease management interventions for health care consumers: a systematic review. PLoS Med.

[33] Berrouiguet S, Baca-García Enrique, Brandt S, Walter M, Courtet P (2016). Fundamentals for Future Mobile-Health (mHealth): A Systematic Review of Mobile Phone and Web-Based Text Messaging in Mental Health. J Med Internet Res.

[34] Shaw R, Bosworth H (2012). Short message service (SMS) text messaging as an intervention medium for weight loss: A literature review. Health Informatics J.

[35] Sibeko G, Temmingh H, Mall S, Williams-Ashman P, Thornicroft G, Susser ES, Lund C, Stein DJ, Milligan PD (2017). Improving adherence in mental health service users with severe mental illness in South Africa: a pilot randomized controlled trial of a treatment partner and text message intervention vs. treatment as usual. BMC Res Notes.

[36] Sims H, Sanghara H, Hayes D, Wandiembe S, Finch M, Jakobsen H, Tsakanikos E, Okocha CI, Kravariti E (2012). Text message reminders of appointments: a pilot intervention at four community mental health clinics in London. Psychiatr Serv.

[37] Lee HY, Koopmeiners JS, Rhee TG, Raveis VH, Ahluwalia JS (2014). Mobile phone text messaging intervention for cervical cancer screening: changes in knowledge and behavior pre-post intervention. J Med Internet Res.

[38] Pereira AAC, Destro JR, Picinin Bernuci M, Garcia LF, Rodrigues Lucena TF (2020). Effects of a WhatsApp-Delivered Education Intervention to Enhance Breast Cancer Knowledge in Women: Mixed-Methods Study. JMIR Mhealth Uhealth.

[39] Whatsapp Inc.

[40] Most popular messaging apps. Statista.

[41] (2010). Microsoft excel. Microsoft Corporation.

[42] (2017). Qualtrics XM.

[43] Hong YW (2017). Efficacy of a Valued Living Program Delivered Through Instant Messaging Application in Promoting Mental Well-being of College Students in Hong Kong [master thesis].

[44] Kabat-Zinn J (2013). Full Catastrophe Living, Revised Edition: How to Cope with Stress, Pain and Illness Using Mindfulness Meditation.

[45] Bastien C (2001). Validation of the Insomnia Severity Index as an outcome measure for insomnia research. Sleep Medicine.

[46] Cincotta AL, Gehrman P, Gooneratne NS, Baime MJ (2010). The effects of a mindfulness-based stress reduction programme on pre-sleep cognitive arousal and insomnia symptoms: a pilot study. Stress and Health.

[47] Gross CR, Kreitzer MJ, Reilly-Spong M, Wall M, Winbush NY, Patterson R, Mahowald M, Cramer-Bornemann M (2011). Mindfulness-based stress reduction versus pharmacotherapy for chronic primary insomnia: a randomized controlled clinical trial. Explore (NY).

[48] Ong JC, Shapiro SL, Manber R (2009). Mindfulness meditation and cognitive behavioral therapy for insomnia: a naturalistic 12-month follow-up. Explore (NY).

[49] Kan KK (2008). Validation of the insomnia severity index, Athens insomnia scale and sleep quality index in adolescent population in Hong Kong. Published online 2008, b4073.

[50] Chung Ka-Fai, Kan Katherine Ka-Ki, Yeung Wing-Fai (2011). Assessing insomnia in adolescents: comparison of Insomnia Severity Index, Athens Insomnia Scale and Sleep Quality Index. Sleep Med.

[51] Nicassio PM, Mendlowitz DR, Fussell JJ, Petras L (1985). The phenomenology of the pre-sleep state: The development of the pre-sleep arousal scale. Behaviour Research and Therapy.

[52] Brislin RW (1986). The wording and translation of research instruments. Field Methods in Cross-Cultural Research.

[53] Morin C, Vallières Annie, Ivers H (2007). Dysfunctional beliefs and attitudes about sleep (DBAS): validation of a brief version (DBAS-16). Sleep.

[54] Chen CW, Jan YW, Yang CM, Lin SC (2009). Dysfunctional beliefs and attitudes about sleep (DBAS): validation of the Chinese version. Archives of Clinical Psychology.

[55] Kelly AM (2001). The minimum clinically significant difference in visual analogue scale pain score does not differ with severity of pain. Emerg Med J.

[56] Herr KA, Spratt K, Mobily PR, Richardson G (2004). Pain intensity assessment in older adults: use of experimental pain to compare psychometric properties and usability of selected pain scales with younger adults. Clin J Pain.

[57] Joyce CRB, Zutshi DW, Hrubes V, Mason RM (1975). Comparison of fixed interval and visual analogue scales for rating chronic pain. Eur J Clin Pharmacol.

[58] McCracken LM (1998). Learning to live with the pain: acceptance of pain predicts adjustment in persons with chronic pain. Pain.

[59] Cheung Michelle N, Ning Michelle Cheung, Wong Tony C M, Ming Tony Wong Chi, Yap Jacqueline C M, Mae Jacqueline Yap Chooi, Chen Phoon P, Ping Chen Phoon (2008). Validation of the Chronic Pain Acceptance Questionnaire (CPAQ) in Cantonese-speaking Chinese patients. J Pain.

[60] Sullivan MJL, Bishop SR, Pivik J (1995). The Pain Catastrophizing Scale: Development and validation. Psychological Assessment.

[61] Yap JC, Lau J, Chen PP, Gin T, Wong T, Chan I, Chu J, Wong E (2008). Validation of the Chinese Pain Catastrophizing Scale (HK-PCS) in patients with chronic pain. Pain Med.

[62] Karlsson J, Persson L, Sjöström L, Sullivan M (2000). Psychometric properties and factor structure of the Three-Factor Eating Questionnaire (TFEQ) in obese men and women. Results from the Swedish Obese Subjects (SOS) study. Int J Obes Relat Metab Disord.

[63] Cappelleri JC, Bushmakin AG, Gerber RA, Leidy NK, Sexton CC, Karlsson J, Lowe MR (2009). Evaluating the Power of Food Scale in obese subjects and a general sample of individuals: development and measurement properties. Int J Obes (Lond).

[64] Lowe MR, Butryn ML, Didie ER, Annunziato RA, Thomas JG, Crerand CE, Ochner CN, Coletta MC, Bellace D, Wallaert M, Halford J (2009). The Power of Food Scale. A new measure of the psychological influence of the food environment. Appetite.

[65] Brown KW, Ryan RM (2003). The benefits of being present: mindfulness and its role in psychological well-being. J Pers Soc Psychol.

[66] Carlson LE, Brown KW (2005). Validation of the Mindful Attention Awareness Scale in a cancer population. J Psychosom Res.

[67] Dharma Drum Institute of Liberal Arts ZEN – research on creative meditation space for ease and mindfulness research (report no. nsc 99-2218-e-655-001-). Dharma Drum Institute of Liberal Arts.

[68] Kroenke K, Spitzer RL, Williams JBW (2001). The PHQ-9: validity of a brief depression severity measure. J Gen Intern Med.

[69] Spitzer RL, Kroenke K, Williams JBW, Löwe Bernd (2006). A brief measure for assessing generalized anxiety disorder: the GAD-7. Arch Intern Med.

[70] Wellbeing measures in primary health care: the DepCare project. World Health Organization.

[71] Henkel V, Mergl Roland, Kohnen Ralf, Maier Wolfgang, Möller Hans-Jürgen, Hegerl Ulrich (2003). Identifying depression in primary care: a comparison of different methods in a prospective cohort study. BMJ.

[72] Newnham EA, Hooke GR, Page AC (2010). Monitoring treatment response and outcomes using the World Health Organization's Wellbeing Index in psychiatric care. J Affect Disord.

[73] Topp CW, Østergaard Søren Dinesen, Søndergaard Susan, Bech P (2015). The WHO-5 Well-Being Index: a systematic review of the literature. Psychother Psychosom.

[74] Mundt JC, Marks IM, Shear MK, Greist JM (2002). The Work and Social Adjustment Scale: a simple measure of impairment in functioning. Br J Psychiatry.

[75] Thandi G, Fear NT, Chalder T (2017). A comparison of the Work and Social Adjustment Scale (WSAS) across different patient populations using Rasch analysis and exploratory factor analysis. J Psychosom Res.

[76] van Ginkel JR, Kroonenberg PM (2014). Analysis of Variance of Multiply Imputed Data. Multivariate Behav Res.

[77] Rubin D (1987). Multiple Imputation for Nonresponse in Surveys. Wiley.

[78] Van Ginkel JR (2010). Investigation of Multiple Imputation in Low-Quality Questionnaire Data. Multivariate Behav Res.

[79] Muthen L, Muthen B (2012). Mplus Version 7 User's Guide.

[80] Day MA, Thorn BE (2016). The mediating role of pain acceptance during mindfulness-based cognitive therapy for headache. Complement Ther Med.

[81] Vickers A, de CA (2000). Why use placebos in clinical trials? A narrative review of the methodological literature. J Clin Epidemiol.

[82] Liddon L, Kingerlee R, Barry JA (2018). Gender differences in preferences for psychological treatment, coping strategies, and triggers to help-seeking. Br J Clin Psychol.

[83] Carlson LE (2018). Uptake of mindfulness‐based interventions: A phenomenon of wealthy white western women?. Clinical Psychology: Science and Practice.

[84] Bodenlos JS, Strang K, Gray-Bauer R, Faherty A, Ashdown BK (2016). Male Representation in Randomized Clinical Trials of Mindfulness-Based Therapies. Mindfulness.

