# Supplementary figures and images for: Delivering Mindfulness-Based Interventions for Insomnia, Pain, and Dysfunctional Eating Through a Text Messaging App: Three Randomized Controlled Trials Investigating the Effectiveness and Mediating Mechanisms
Source: J Med Internet Res. 2022 May 3;24(5):e30073. doi: 10.2196/30073 (PMC9115660; doi:10.2196/30073)

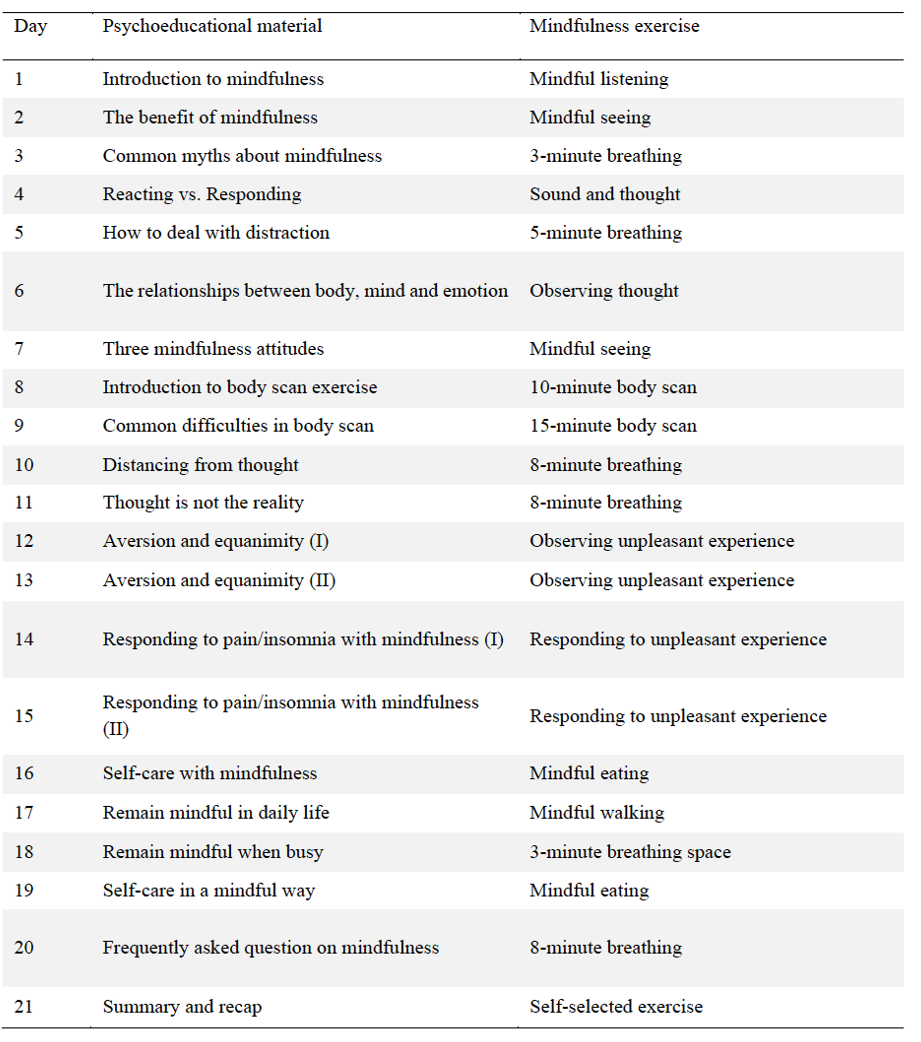

Supplement: Multimedia Appendix 1 [file jmir_v24i5e30073_app1.png]

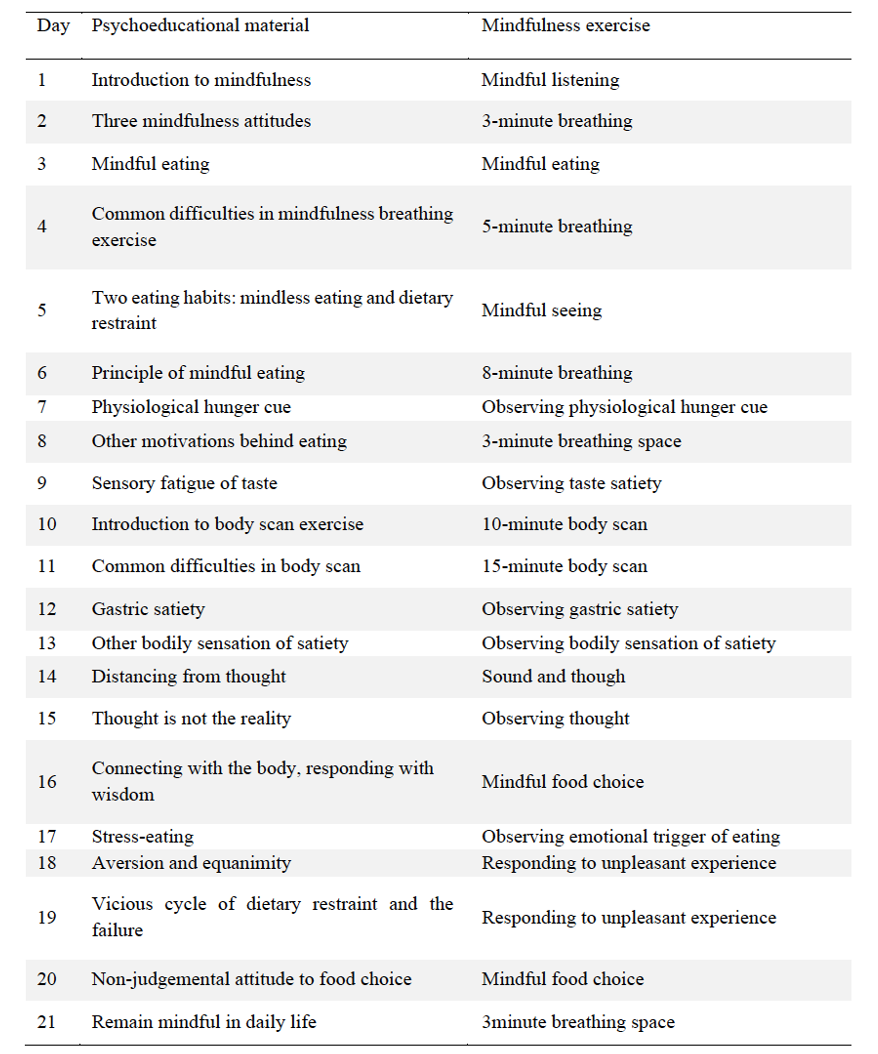

Supplement: Multimedia Appendix 2 [file jmir_v24i5e30073_app2.png]

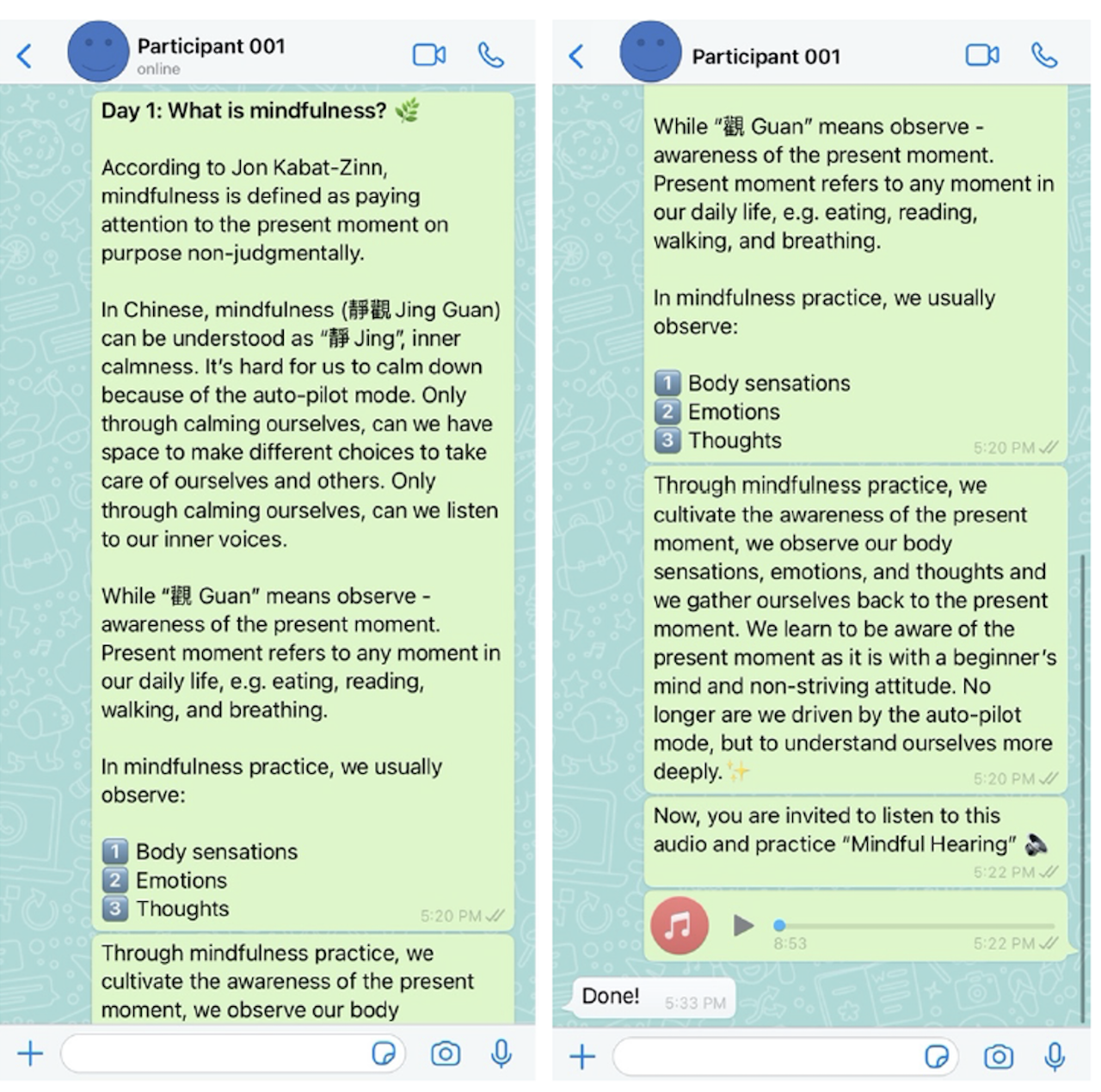

Supplement: Multimedia Appendix 3 [file jmir_v24i5e30073_app3.png]
